# Amino acid availability acts as a metabolic rheostat to determine the magnitude of ILC2 responses

**DOI:** 10.1084/jem.20221073

**Published:** 2022-12-26

**Authors:** Suzanne H. Hodge, Maria Z. Krauss, Irem Kaymak, James I. King, Andrew J.M. Howden, Gordana Panic, Richard K. Grencis, Jonathan R. Swann, Linda V. Sinclair, Matthew R. Hepworth

**Affiliations:** 1 Lydia Becker Institute of Immunology and Inflammation, University of Manchester, Manchester, UK; 2 Division of Infection, Immunity and Respiratory Medicine, School of Biological Sciences, Faculty of Biology, Medicine and Health, Manchester Academic Health Science Centre, University of Manchester, Manchester, UK; 3 Cell Signalling and Immunology Division, School of Life Sciences, University of Dundee, Dundee, UK; 4 Division of Integrative Systems Medicine and Digestive Diseases, Imperial College London, South Kensington, UK; 5 School of Human Development and Health, Faculty of Medicine, University of Southampton, Southampton, UK

## Abstract

Group 2 innate lymphoid cells (ILC2) are functionally poised, tissue-resident lymphocytes that respond rapidly to damage and infection at mucosal barrier sites. ILC2 reside within complex microenvironments where they are subject to cues from both the diet and invading pathogens—including helminths. Emerging evidence suggests ILC2 are acutely sensitive not only to canonical activating signals but also perturbations in nutrient availability. In the context of helminth infection, we identify amino acid availability as a nutritional cue in regulating ILC2 responses. ILC2 are found to be uniquely preprimed to import amino acids via the large neutral amino acid transporters *Slc7a5* and *Slc7a8*. Cell-intrinsic deletion of these transporters individually impaired ILC2 expansion, while concurrent loss of both transporters markedly impaired the proliferative and cytokine-producing capacity of ILC2. Mechanistically, amino acid uptake determined the magnitude of ILC2 responses in part via tuning of mTOR. These findings implicate essential amino acids as a metabolic requisite for optimal ILC2 responses within mucosal barrier tissues.

## Teaser

Here, Hodge et al. demonstrate large neutral amino acid transporters regulate the magnitude of ILC2 responses. Amino acid uptake controls ILC2 expansion in part via mTOR. These findings expand understanding of nutrient regulation of innate immunity at mucosal barrier sites.

## Graphical Abstract

**Figure:**
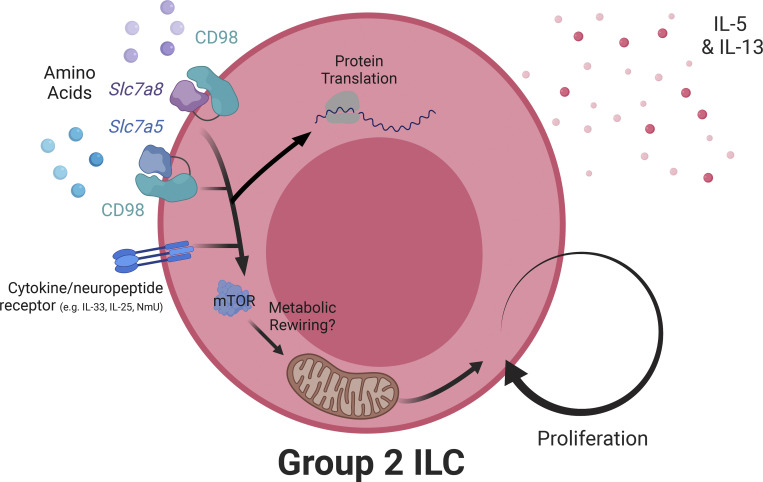

## Introduction

Type 2 immune responses are specialized to induce effector mechanisms that mediate protective immunity to large extracellular helminth parasites, which invade and inhabit barrier tissues (Sorobetea et al., 2018; Harris and Loke, 2017). Indeed, helminth infections have been postulated to be the major evolutionary driver of type 2 immune system, although the precise factors that regulate the magnitude and quality of type 2 immune cell responses remain incompletely defined. Chronic helminth infections are associated with significant morbidity—including malnutrition—potentially due to competition with the host for metabolic resources, which in turn can have potent immunomodulatory consequences (van der Zande et al., 2019; Moyat et al., 2019). Indeed, an emerging body of evidence suggests the mammalian immune system is primed to sense and utilize nutrients and metabolites derived directly from the diet or produced by the commensal microbiota or pathogenic organisms (Kedia-Mehta and Finlay, 2019; Buck et al., 2017). Moreover, gastrointestinal helminth infections are associated with alterations in both the microbiota and dietary nutrient availability (van der Zande et al., 2019; Moyat et al., 2019; Jenkins et al., 2017; Ramanan et al., 2016; Zaiss et al., 2015).

Group 2 innate lymphoid cells (ILC2) are transcriptionally and functionally poised effector immune cells found primarily at mucosal barrier sites, and which respond rapidly during the early phases of helminth infection by robustly producing the effector cytokines IL-5 and IL-13 (Klose and Artis, 2016; Vivier et al., 2018). Alarmin signals—including IL-25, IL-33, and thymic stromal lymphopoietin, and released by non-hematopoietic cells in response to tissue damage—act in concert with cues from tissue-resident neurons and glial cells to induce rapid proliferation and expansion of ILC2 and elicit protective responses such as eosinophilia, goblet cell hyperplasia, epithelial cell extrusion, and smooth muscle hypercontractility (Klose and Artis, 2016; Vivier et al., 2018). In addition, it is increasingly appreciated that ILC2 can sense and respond to changes in the abundance and availability of dietary and microbially derived metabolites, including vitamin A–derived retinoic acid (Spencer et al., 2014), aryl-hydrocarbon receptor ligands (Li et al., 2018), short chain fatty acids (Lewis et al., 2019), succinate (Schneider et al., 2018; Nadjsombati et al., 2018), and lipids (Karagiannis et al., 2020), suggesting that ILC2 are poised to sense not only tissue-associated danger signals but also the broader metabolic milieu of mucosal tissues (Shi et al., 2021).

In addition, micronutrients are key determinants of immune effector function through their capacity to provide fundamental substrates for production of the energy and biomass needed to fuel proliferation and protein translation (Kedia-Mehta and Finlay, 2019; Buck et al., 2017). Indeed, the ability of ILC2 to mount an effective and appropriate response to challenge has been shown to be dependent on the ability to appropriately engage cell-intrinsic metabolic pathways to catabolize glucose, fatty acids, and arginine (Karagiannis et al., 2020; Wilhelm et al., 2016; Monticelli et al., 2016). Despite these advances, it remains unclear whether changes in the availability of metabolites occur during infection that may determine the quality and magnitude of ILC2 responses. Moreover, the precise nature of metabolic cues that modulate ILC2 responses and underpin their rapid and innate effector functions remain incompletely defined.

Here, we identify amino acid availability as a critical rheostat of ILC2 responses. Strikingly—and unlike other tissue-resident immune cells at steady state—ILC2 were found to express multiple solute carrier-encoded transporters that act to ensure ILC2 are prepoised to acquire essential amino acids from the environment. Notably, loss of these transporters, either individually or concurrently, impaired the ILC2 response. This was found to be in part through their ability to tune ILC2 metabolic fitness and mammalian target of rapamycin (mTOR) pathway activation. Together, these findings suggest that ILC2 are metabolically primed to facilitate rapid expansion following activation by alarmins or in the context of helminth infection.

## Results

### Amino acid availability impacts type 2 immunity during helminth infection

**Figure 1.**
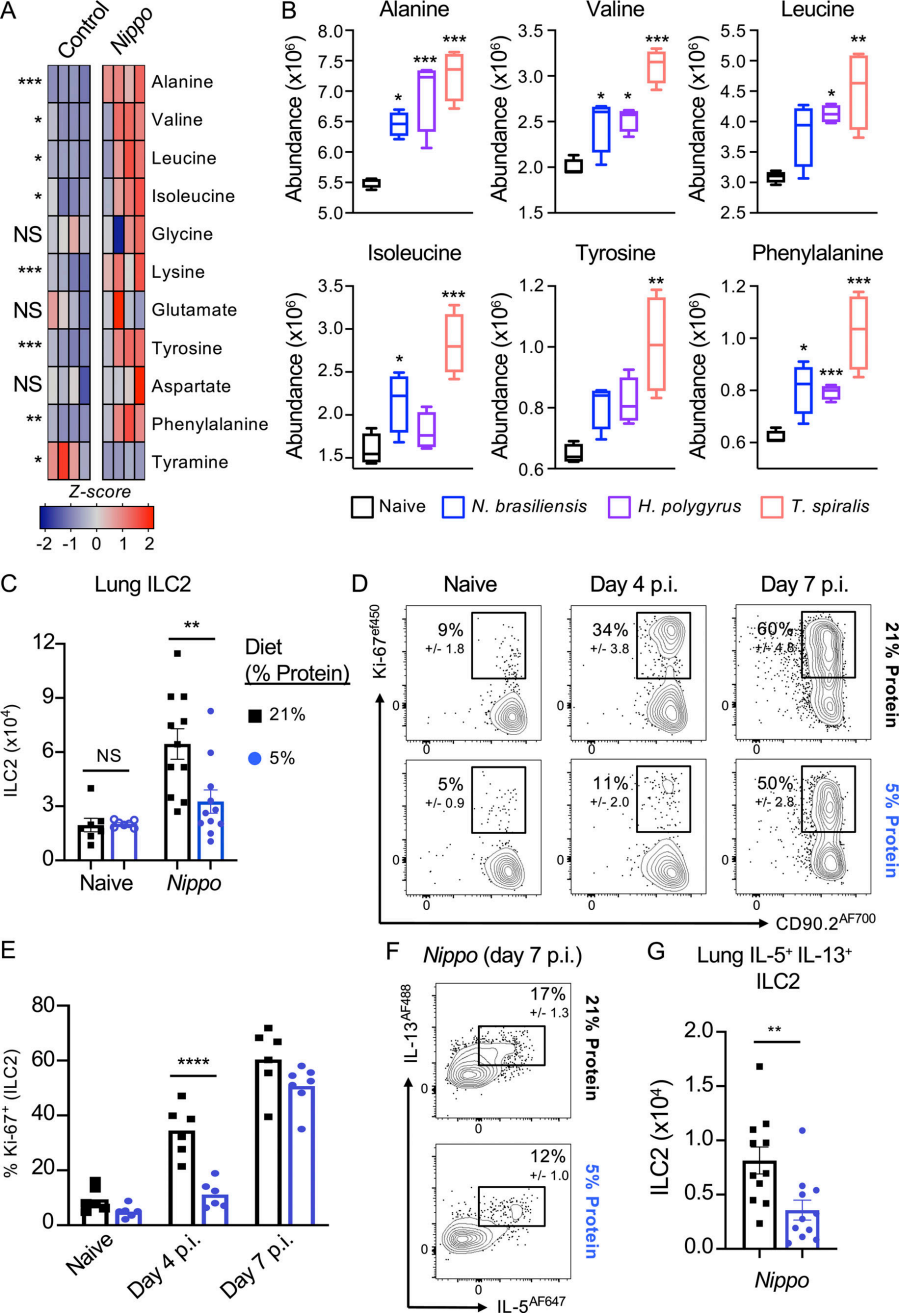
**Metabolite and dietary factors influence innate type 2 response to helminth infection. (A)** Relative levels of fecal amino acids and amino acid–related metabolites in control and day 7 post-infection (p.i.) *N. brasiliensis*–infected C57BL/6 mice (*n* = 4 mice per group, representative of two independent experiments, data shows *z*-scores). **(B)** Relative abundance of selected amino acids in naive mice or mice infected with *N. brasiliensis* (*Nippo*; day 7 p.i., blue), *H. polygyrus* (day 7 p.i., purple), or *T. spiralis* (day 7 p.i., pink). *n* = 4 mice per group, representative of one experiment, data show relative abundance. **(C–G)** (C) Numbers of ILC2 in naive or *N. brasiliensis*–infected (day 7 p.i.) mice, (D) frequency and (E) number of Ki-67^+^ ILC2 on days 4 and 7 after *N. brasiliensis* infection, and (F) frequency and (G) number of IL-5 and IL-13 producing ILC2 on day 7 after *N. brasiliensis* infection in C57BL/6 mice fed a normal (21%) or low (5%) protein diet. *n =* 6–11 mice per group, pooled data from two independent experiments. ILC2 gated as Live CD45^+^ Lineage negative (CD3^−^ CD5^−^ NK1.1^−^ B220^−^ CD11b^−^ CD11c^−^) CD127^+^ CD90.2^+^ GATA-3^hi^ KLRG1^+^ cells. Data are shown as individual values or mean ± SEM. Statistical tests performed: (A, B, C, and E) one-way ANOVA with multiple comparisons, (G) Mann–Whitney *t* test. * P < 0.05, ** P < 0.01, *** P < 0.001.

**Figure S1.**
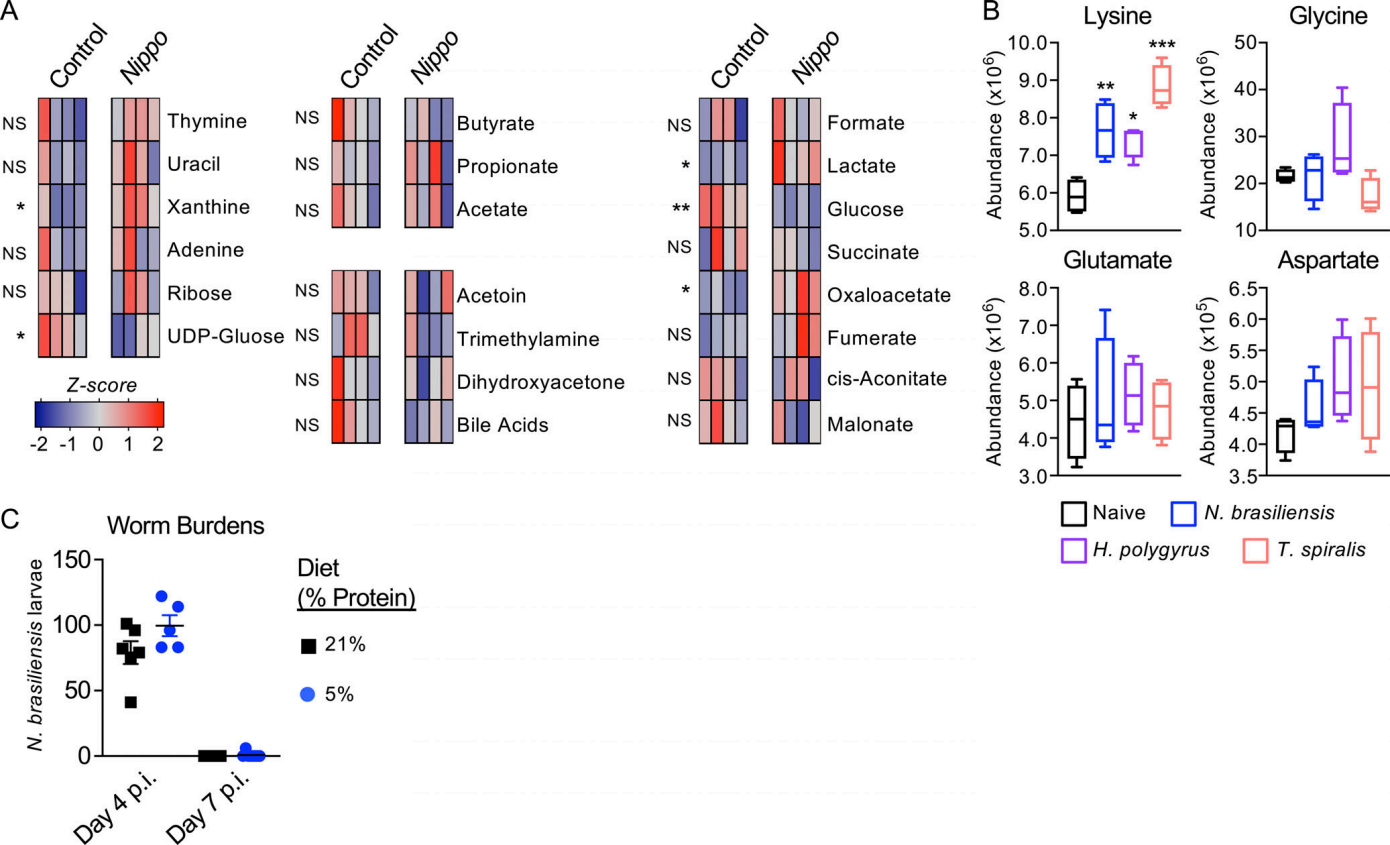
**Changes in metabolite and dietary factors following helminth infection. (A)** Relative abundance of fecal metabolites in control and day 7 post infection (p.i.) *N. brasiliensis* (*Nippo*)–infected C57BL/6 mice (*n* = 4 mice per group, representative of two independent experiments, data shows *z*-scores). **(B)** Relative abundance of amino acids (related to Fig. 1 A) in naive mice or mice infected with *N. brasiliensis* (day 7 p.i., blue), *H. polygyrus* (day 7 p.i., purple), or *T. spiralis* (day 7 p.i., pink). *n* = 4 mice per group, representative of one experiment, data shows relative abundance. **(C)** Small intestinal worm burdens from *N. brasiliensis*–infected mice fed 21% of 5% protein diet at day 4 and day 7 p.i.. Data representative of *n =* 5–6 mice per group per timepoint and two independent experiments. Data are shown as individual values or mean ± SEM. Statistical tests performed: (A and B) one-way ANOVA with multiple comparisons. * P < 0.05, ** P < 0.01, *** P < 0.001.

To identify environmental and metabolic cues that could impact innate type 2 immune responses in the context of helminth infection, we infected mice with *Nippostrongylus brasiliensis* for 7 d and performed unbiased metabolomic analysis on feces of infected and control animals (Fig. 1 A). Using this approach, we identified a total of 32 unique molecules, of which 13 were found to differ significantly (P < 0.05). We identified changes in the relative abundance of several metabolites following infection, including a relative decrease in glucose and increase in lactate in the feces of infected mice, whereas no consistent differences were observed in the abundance of common microbial metabolites, such as short-chain fatty acids (Fig. S1 A). Notably, we detected increases in the relative abundance of a number of amino acids following infection, including alanine, valine, leucine, and isoleucine, among others. Many of the amino acids found to be increased are “essential” amino acids that cannot be synthesized by mammalian cells, and instead must be acquired from dietary and environmental sources (Fig. 1, A and B; and Fig. S1 B). Comparable analysis of mice infected with other small intestinal–dwelling helminths, specifically *Heligmosomoides polygyrus* and *Trichinella spiralis*, yielded similar changes in fecal amino acid abundance, albeit to different degrees (Fig. 1 B and Fig. S1 B).

Given the previously reported impact of nutrient availability on ILC2 responses (Karagiannis et al., 2020; Wilhelm et al., 2016; Monticelli et al., 2016; Spencer et al., 2014), we hypothesized that an altered abundance of amino acids in the gastrointestinal tract may impact the quality or magnitude of a protective innate immune response during helminth infection. To test this, we fed mice a diet that was relatively low in protein (5% energy from protein), which has previously been shown to reduce both tissue and systemic amino acid availability (Yap et al., 2020), and compared to mice fed a control diet (21% energy from protein; comparable with normal chow used in these studies). We focused our analysis on the lung—a tissue through which *N*. *brasiliensis* migrates during the first days of infection, inducing significant tissue damage and eliciting a potent ILC2 response. Mice fed a 5% protein diet exhibited reduced accumulation of ILC2 numbers by day 7 after infection (Fig. 1 C), which was associated with a delayed proliferative response as compared with 21% protein diet–fed mice (Fig. 1, D and E). ILC2 exhibited a moderate reduction in intrinsic capacity to produce IL-5 and IL-13 in response to infection (Fig. 1 F); however, when coupled with decreased cellularity, this led to an overall reduction in the number of cytokine-producing ILC2 (Fig. 1 G). Nonetheless, worm expulsion still occurred in animals fed a low-protein diet (Fig. S1 C), suggesting the ILC2 response generated was ultimately sufficient to mediate protective immunity in this context. Thus, these data indicated that altering the availability of amino acids may modulate the quality and magnitude of the ILC2 response.

### ILC2 are poised for amino acid uptake

**Figure 2.**
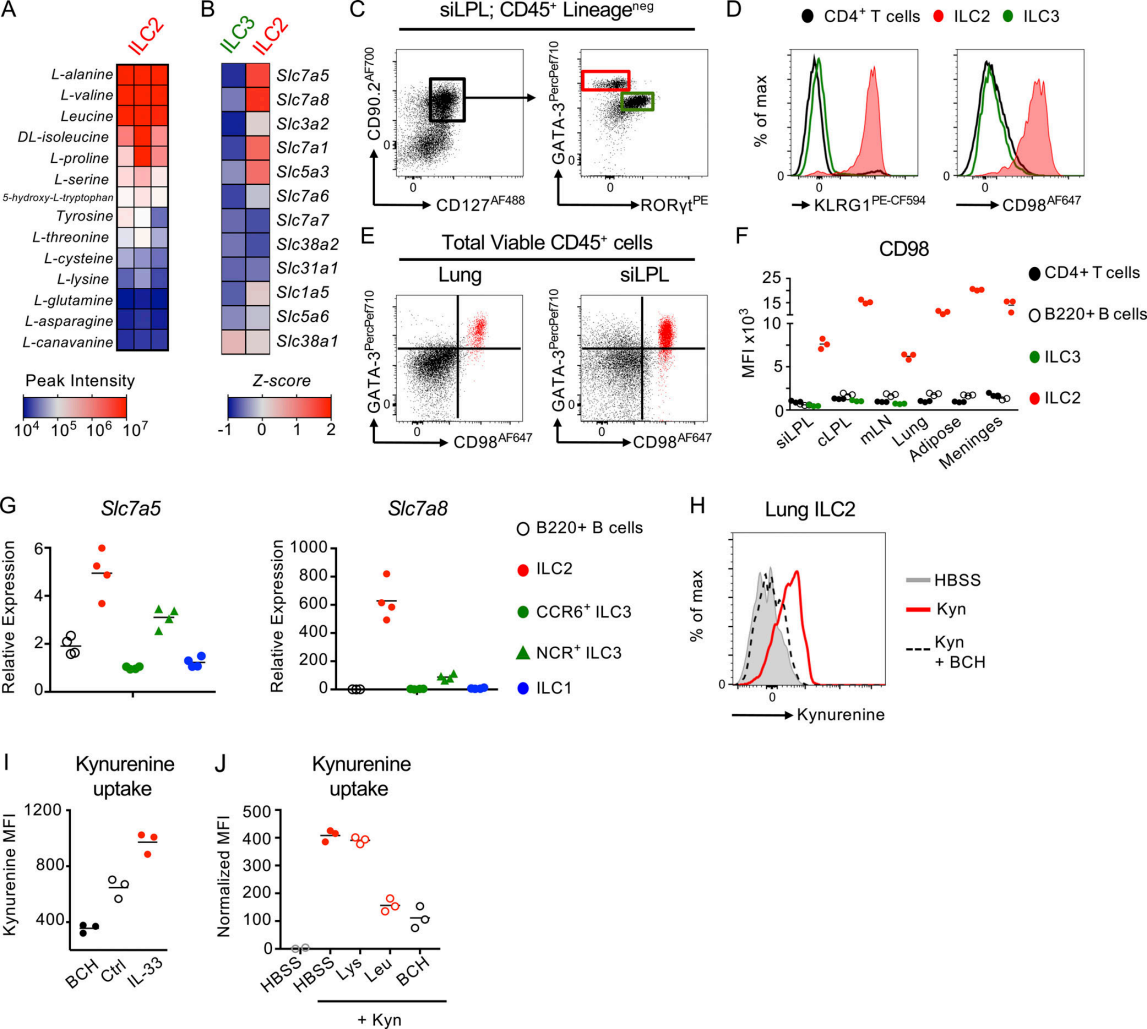
**ILC2 are preferentially poised to import large neutral amino acids. (A)** Analysis of the intracellular amino acid content of ILC2 sort-purified from IL-33–treated mice (*n =* 3 independent replicates of cells pooled from two mice and representative of two independent experiments). **(B)** Comparison of mean expression of amino acid transporter-associated genes in ILC2 and CCR6^+^ ILC3 (ILC3) from public data (http://www.immgen.org [Robinette et al., 2015]). **(C and D)** (C) Representative gating and (D) surface expression of KLRG1 and CD98 on CD4^+^ T cells (black), ILC2 (red), and ILC3 (green) from small intestinal lamina propria (siLPL). **(E)** Representative flow plots demonstrating co-expression of GATA-3 and CD98 in lung and siLPL amongst total CD45^+^ cells. **(F)** Expression of CD98 on CD4^+^ T cells (black), B220^+^ B cells (white), ILC3 (green), and ILC2 (red) in siLPL, colon lamina propria (cLPL), mLN, lung, white adipose tissue (fat), and meninges. **(C–F)** *n* = 3 and representative of at least three independent experiments. **(G)** Relative expression of *Slc7a5* and *Slc7a8* in small intestinal ILC subsets and B cells, normalized to CCR6^+^ ILC3 (*n =* 4 per group and representative of at least two independent experiments). **(H)** Representative histogram of Kynurenine uptake in lung ILC2 incubated for 5 min with either HBSS alone (negative control), 200 μM Kynurenine (Kyn) or Kynurenine plus 10 mM BCH. **(I)** Kynurenine uptake in ILC2 from naive (Ctrl) or IL-33–treated mice or incubated with Kynurenine and BCH (*n =* 3 per group and representative of two independent experiments). **(J)** Kynurenine uptake in lung ILC2 in the presence or absence of excess (5 mM) Lysine (Lys), Leucine (Leu), or 10 mM BCH (*n* = 3 replicates per condition, and representative of at least three independent experiments). ILC2 gated as in C or for G–J as Live CD45^+^ Lineage negative (CD3^−^ CD5^−^ NK1.1^−^ B220^−^ CD11b^−^ CD11c^−^) CD127^+^ CD90.2^+^ ST2^+^ KLRG1^+^ cells.

Our data suggested the induction of ILC2 responses may be sensitive to changes in the abundance of essential amino acids derived from dietary intake. Intriguingly, we observed that sort-purified ILC2 isolated from IL-33–treated mice exhibited a relative enrichment within their intracellular contents for many of the same amino acids, including alanine, valine, leucine, and isoleucine (Fig. 2 A). To determine the underpinning molecular machinery through which alterations in amino acid abundance could potentially affect ILC2, we examined published bulk RNA sequencing (seq) data (Robinette et al., 2015) to analyze the expression of a range of solute carrier genes known to act as surface amino acid transporters in ILC2 in comparison with CCR6^+^ ILC3 (ILC3; Fig. 2 B). We observed that ILC2, but not ILC3, constitutively expressed high levels of multiple solute carriers, most notably *Slc3a2*, *Slc7a5*, and *Slc7a8*, known to encode for large neutral amino acid transporter chains (Fig. 2 B). *Slc3a2* encodes the protein CD98—a chaperone molecule and heavy chain subunit—that heterodimerizes with other solute carriers to form active amino acid transporters, and ILC2 were also enriched for the CD98 binding partners *Slc7a5* and *Slc7a8*, which together form the surface amino acid transporters LAT1 and LAT2, respectively. In contrast, expression of other CD98 binding partners, such as *Slc7a6*, *Slc7a7*, *Slc43a1*, and *Slc43a2* were either not enriched in ILC2 or not detected in the data set (Fig. 2 B; Immgen database [Robinette et al., 2015]). LAT1 and LAT2 primarily transport a range of amino acid substrates with overlapping specificity to those observed to be enriched in both the feces of helminth-infected mice and enriched within the intracellular content of ILC2 (Fig. 1, A and B; and Fig. 2 A; Kelly and Pearce, 2020; Kantipudi et al., 2020; Lin et al., 2015; Pineda et al., 1999), suggestive of a possible role for these transporters in ILC2 responses.

**Figure S2.**
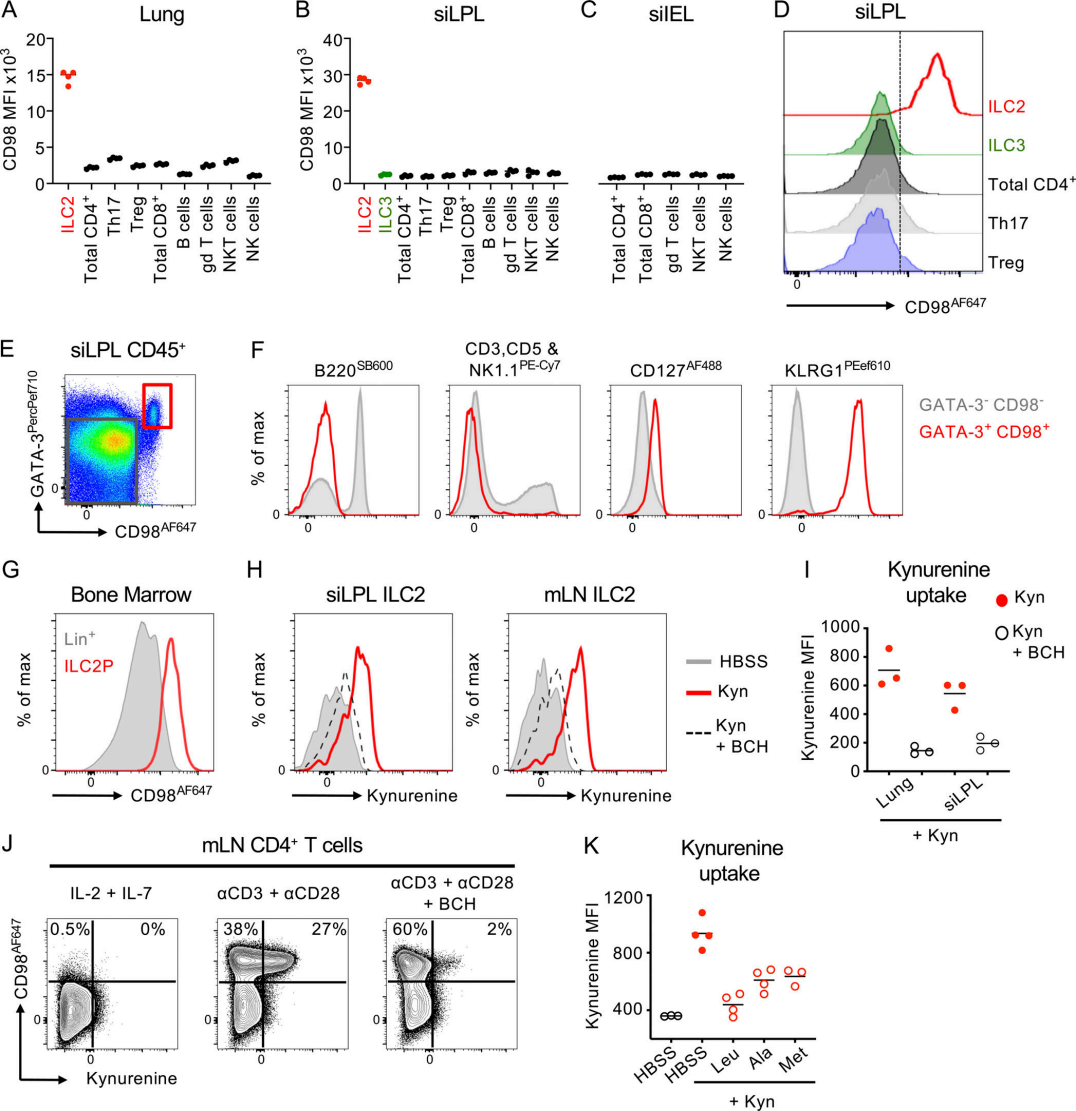
**Amino acid transport in ILC2 and other tissue resident lymphocytes. (A–D)** CD98 MFI in tissue-resident lymphocytes isolated from naive mice tissues. (A) Lung, (B) small intestinal lamina propria (siLPL), (C) small intestinal intraepithelial lymphocytes (siIEL). **(D)** Representative flow plot comparing ILC2 to ILC3 and tissue-resident T cell subsets in the siLPL. Cells gated as Live CD45^+^ and further subgated as: Lineage negative (CD3^−^ CD5^−^ NK1.1^−^ B220^−^) CD127^+^ CD90.2^+^ GATA-3^hi^ KLRG1^+^ - ILC2; Lineage negative (CD3^−^ CD5^−^ NK1.1^−^ B220^−^) CD127^+^ CD90.2^+^ GATA-3^lo^ RORγt^+^ - ILC3; CD3^+^ CD5^+^ Tcrβ^+^ CD4^+^ - total CD4^+^ T cells; CD3^+^ CD5^+^ CD4^+^ RORγt^+^ - Th17; CD3^+^ CD5^+^ CD4^+^ - FoxP3^+^ Treg; CD3^+^ CD5^+^ Tcrβ^+^ CD8^+^ - CD8^+^ T cells; CD3^+^ CD5^+^ Tcrγδ^+^ - gd T cells; CD3^+^ CD5^+^ Tcrγδ^−^ CD27^±^ CD1d (PBS-57)-Tet^+^ - natural killer T (NKT) cells; CD3^−^ CD5^−^ B220^−^ NK1.1^+^ - NK cells. **(A–D)** *n = 4* mice per group and representative of two independent experiments. **(E and F)** Enrichment of ILCs by CD98^+^ GATA-3^+^ gating from total CD45^+^ lymphocytes (*n =* 4–5 mice per group, representative of at least three independent experiments). **(G)** CD98 expression on CD127^+^ CD25^+^ ST2^+^ ILC2 progenitors (ILC2P; red) and Lineage positive bone marrow cells (Lin^+^; gray). *n* = 4 mice per group and representative of two independent experiments. **(H)** Representative histogram of Kynurenine uptake in small intestinal or mLN ILC2 incubated for 5 min with 200 μM Kynurenine (Kyn) or Kynurenine plus 10 mM BCH. **(I)** Quantification of *n = *3 per group, representative of two independent experiments. **(J)** Exemplar CD98 expression and Kynurenine uptake in CD4^+^ T cells from the mLN cultured with either rIL-2 and rIL-7 alone, anti-CD3 and anti-CD28 monoclonal antibodies, or anti-CD3 and anti-CD28 plus BCH (*n* = 2–4 replicates per group, representative of two independent experiments). **(K)** Kynurenine uptake in lung ILC2 in the presence or absence of excess Leucine (Leu), Alanine (Ala), or Methionine (Met). *n* = 3–4 replicates per condition, and representative of two independent experiments.

Consistent with the transcriptional data, we could detect constitutively elevated steady-state expression of CD98 protein on the cell surface of ILC2—but not ILC3, CD4^+^ T cells, or B220^+^ B cells—in a wide range of tissues (Fig. 2, C–F). Indeed, we observed CD98 to be relatively restricted to ILC2 across a range of tissue-resident innate and adaptive lymphocytes in the lung and intestinal tract at steady state (Fig. S2, A–D). In line with this, we found a combination of GATA-3 and CD98 alone was sufficient to identify ILC2 amongst total CD45^+^ cells without prior lineage exclusion or pregating on classical ILC-associated markers (CD127, CD90.2), further indicating the preferentially elevated expression of CD98 by ILC2 amongst mucosal-resident lymphocytes (Fig. 2 E and Fig. S2, E and F), Additionally, CD98 could also be detected on bone marrow ILC2 precursors (Fig. S2 G). To validate whether surface CD98 expression on ILC2 was indicative of LAT activity, we confirmed expression of both *Slc7a5* and *Slc7a8* by RT-PCR in sort-purified ILC2 from the small intestine in comparison with control populations (Fig. 2 G). We then utilized a previously reported assay that utilizes the autofluorescent properties of the tryptophan metabolite kynurenine as a proxy of LAT transporter activity and amino acid uptake (Sinclair et al., 2018). Uptake of kynurenine was detected in naive ILC2 from the lung, small intestine, and mesenteric lymph node, could be inhibited by coculture with the LAT-inhibitor 2-Aminobicyclo-(2,2,1)-heptane-2-carboxylic acid (BCH; Fig. 2 H and Fig. S2, H and I), and was found to be enhanced in ILC2 from IL-33 treated mice (Fig. 2 I). In contrast with naive ILC2, kynurenine uptake in CD4^+^ T cells required TCR engagement to both upregulate surface CD98 and actively take up kynurenine (Fig. S2 J), in line with previous findings (Sinclair et al., 2018; Sinclair et al., 2013). Moreover, uptake of kynurenine by ILC2 was reduced by competition with excess levels of the high-affinity LAT substrates leucine and methionine, as well as alanine (a high-affinity substrate of Slc7a8), but not lysine—which is not transported by Slc7a5 or Slc7a8 but rather by related y+LAT family members (Fig. 2 J and Fig. S2 K; Kanai et al., 1998; Park et al., 2005; Rossier et al., 1999). Together, these findings indicate that ILC2 are preferentially poised to import amino acids via the surface large neutral amino acid transporters *Slc7a5* (LAT1) and *Slc7a8* (LAT2).

### Cell-intrinsic deletion of *Slc7a5* or *Slc7a8* impairs ILC2 expansion

**Figure 3.**
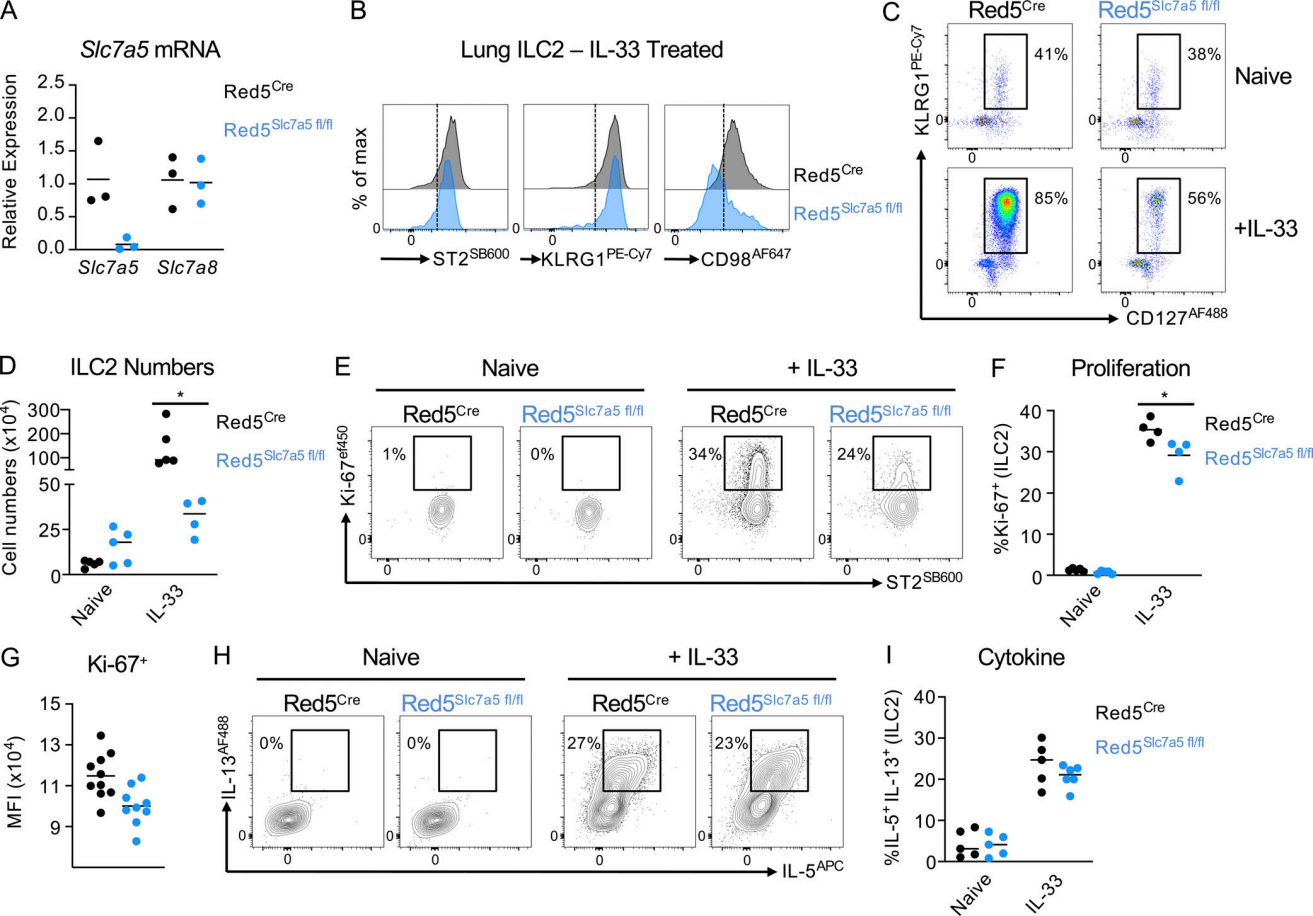
***Slc7a5*/LAT1 regulates the magnitude of ILC2 expansion following activation. (A)** Validation of *Slc7a5* deletion in Red5^Cre^ x *Slc7a5*^fl/fl^ mice (*n =* 3 technical replicates of sort-purified ILC2 pooled from the lungs of two to three IL-33–treated mice per replicate, representative of two independent experiments). **(B)** Representative histograms of ST2, KLRG1, and CD98 in Red5^Cre^ control and Red5^Cre^ x *Slc7a5*^fl/fl^ mice (*n* = 3–4 mice per group and representative of at least three independent experiments). **(C and D)** (C) Frequencies and (D) numbers of KLRG1+ CD127+ ILC2 (pregated on CD45^+^ Lineage negative cells) in the lungs of naive or IL-33–treated Red5^Cre^ control and Red5^Cre^ x *Slc7a5*^fl/fl^ mice. *n* = 4–5 mice per group, representative of at least three independent experiments. **(E–G)** (E) Representative flow plots, (F) quantification, and (G) mean fluorescent intensity (MFI) of Ki-67 expression in ILC2 from control and IL-33–treated Red5^Cre^ control and Red5^Cre^ x *Slc7a5*^fl/fl^ mice. E and F, *n* = 4 mice per group, representative of three independent experiments; G, *n =* 9–10 per group and pooled from two independent experiments. **(H and I)** (H) Representative flow plots and (I) quantification of IL-5 and IL-13 producing ILC2 from control and IL-33–treated Red5^Cre^ control and Red5^Cre^ x *Slc7a5*^fl/fl^ mice. *n* = 5–6 mice per group, representative of two independent experiments. ILC2 gated as in Fig. S3 A. Data shown as individual values and mean ± SEM. Statistical tests performed: (D and F) one-way ANOVA with multiple comparisons. * P < 0.05.

**Figure S3.**
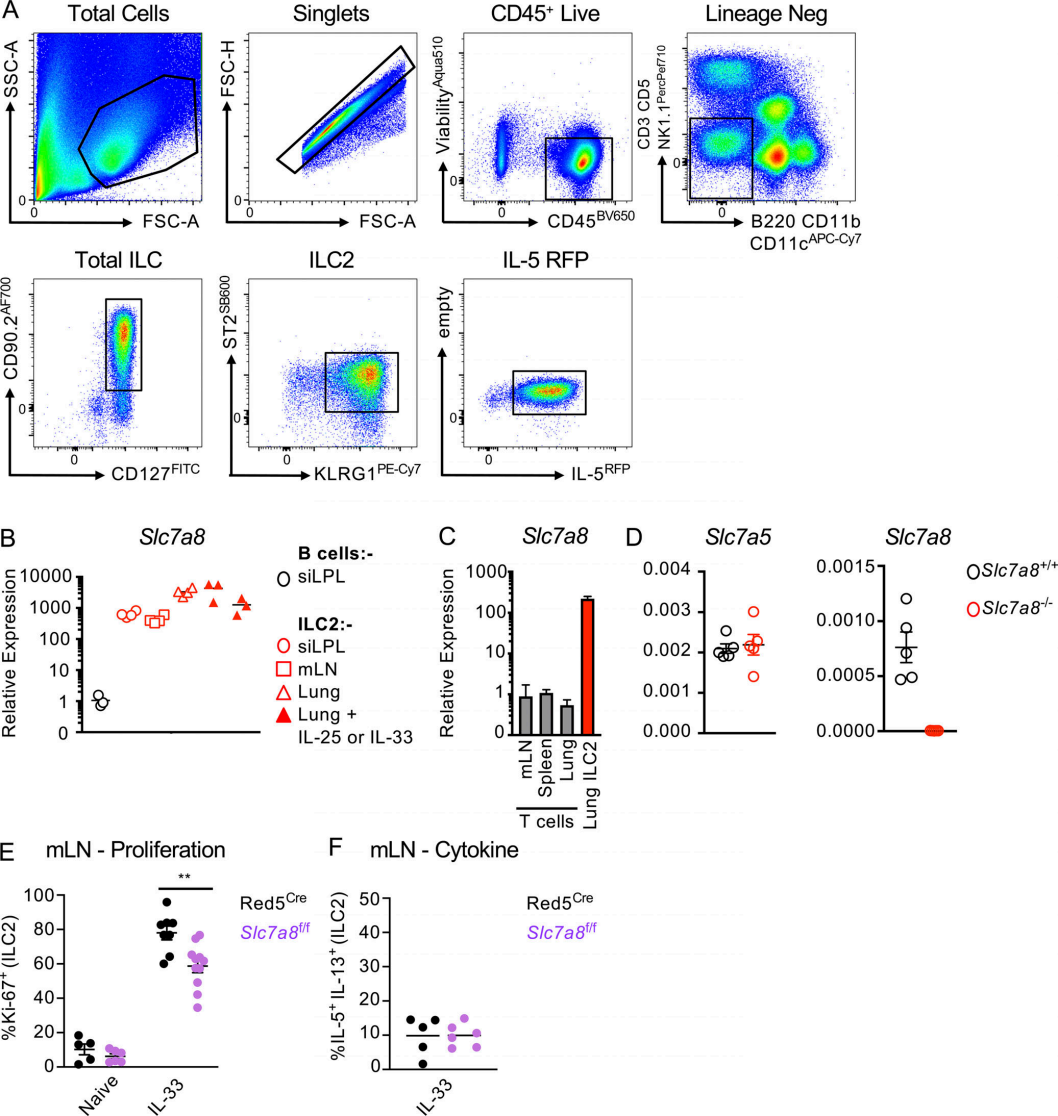
**Selective *Slc7a8* expression in ILC2 and validation of deletion. (A)** Representative gating strategy for ILC2 in Red5^Cre^ and associated flox mice. **(B)** Relative expression of *Slc7a8* in sort purified ILC2 from the small intestinal lamina propria (siLPL), mLN, lung, or lung from mice previously treated with IL-25 or IL-33, normalize to sort-purified B cells from the Peyer’s patches and siLPL (*n* = 3–4 technical replicates pooled from two to three mice per group and representative of two to three independent experiments). **(C)** Relative expression of *Slc7a8* in sort-purified lung ILC2, in comparison to bulk CD3^+^ T cells sort-purified from the mLN, spleen, or lung (*n* = 4 technical replicates pooled from two to three mice per group and representative of two independent experiments). **(D)** Expression of *Slc7a5* and *Slc7a8* in naive sort-purified ILC2 from the lungs of *Slc7a8*^+/+^ or *Slc7a8*^−/−^ mice (*n* = 5 technical replicates pooled from two to three mice per group and representative of a single experiment). **(E and F)** (E) Quantification of Ki-67^+^ ILC2 and (F) IL-5^+^ IL-13^+^ ILC2 from the mLN of Red5^Cre^ and Red5^Cre^ x *Slc7a8*^fl/fl^ mice. Data representative of *n =* 5–10 mice per group, pooled from two independent experiments. Data shown as individual values or mean ± SEM. Statistical tests performed: (E) one-way ANOVA with multiple comparisons ** P < 0.01.

As ILC2 were found to preferentially express CD98 along with two distinct partner chains *Slc7a5* (LAT1) and *Slc7a8* (LAT2), we next aimed to determine the role of these transporters during an ILC2 response. First, we generated mice with a conditional deletion of *Slc7a5* in ILC2 by crossing Red5^Cre^ mice (Nussbaum et al., 2013) with *Slc7a5*^fl/fl^ mice (Sinclair et al., 2013; Fig. 3 A) and determined the effect on ILC2 responses following activation (representative gating strategy; Fig. S3 A). ILC2 from IL-33–treated Red5^Slc7a5 fl/fl^ mice exhibited comparable expression of ST2 and KLRG1 as compared with Red5^Cre^ control animals, but had a clear reduction in surface CD98 expression (Fig. 3 B). Technical limitations precluded our ability to isolate viable lymphocytes from the small intestine of animals in which type 2 immune responses had been elicited, thus we focused our analyses on the lung. Notably, while ILC2 frequencies at steady state were comparable between Red5^Cre^ and Red5^Slc7a5 fl/fl^ mice, ILC2 lacking cell-intrinsic *Slc7a5* expression demonstrated a clear defect in expansion following in vivo activation with IL-33 (Fig. 3, C and D). This correlated with a reduced percentage of cells expressing Ki-67 and notably, consistently reduced intensity of Ki-67 staining amongst positive cells (Fig. 3, E–G). In contrast, ILC2 exhibited comparable frequencies of IL-5 and IL-13 positive cells in the absence of *Slc7a5* (Fig. 3, H and I), suggesting disruption of a single ILC2-intrinsic amino acid transporter may largely perturb the magnitude but not the effector capacity of ILC2 following activation.

**Figure 4.**
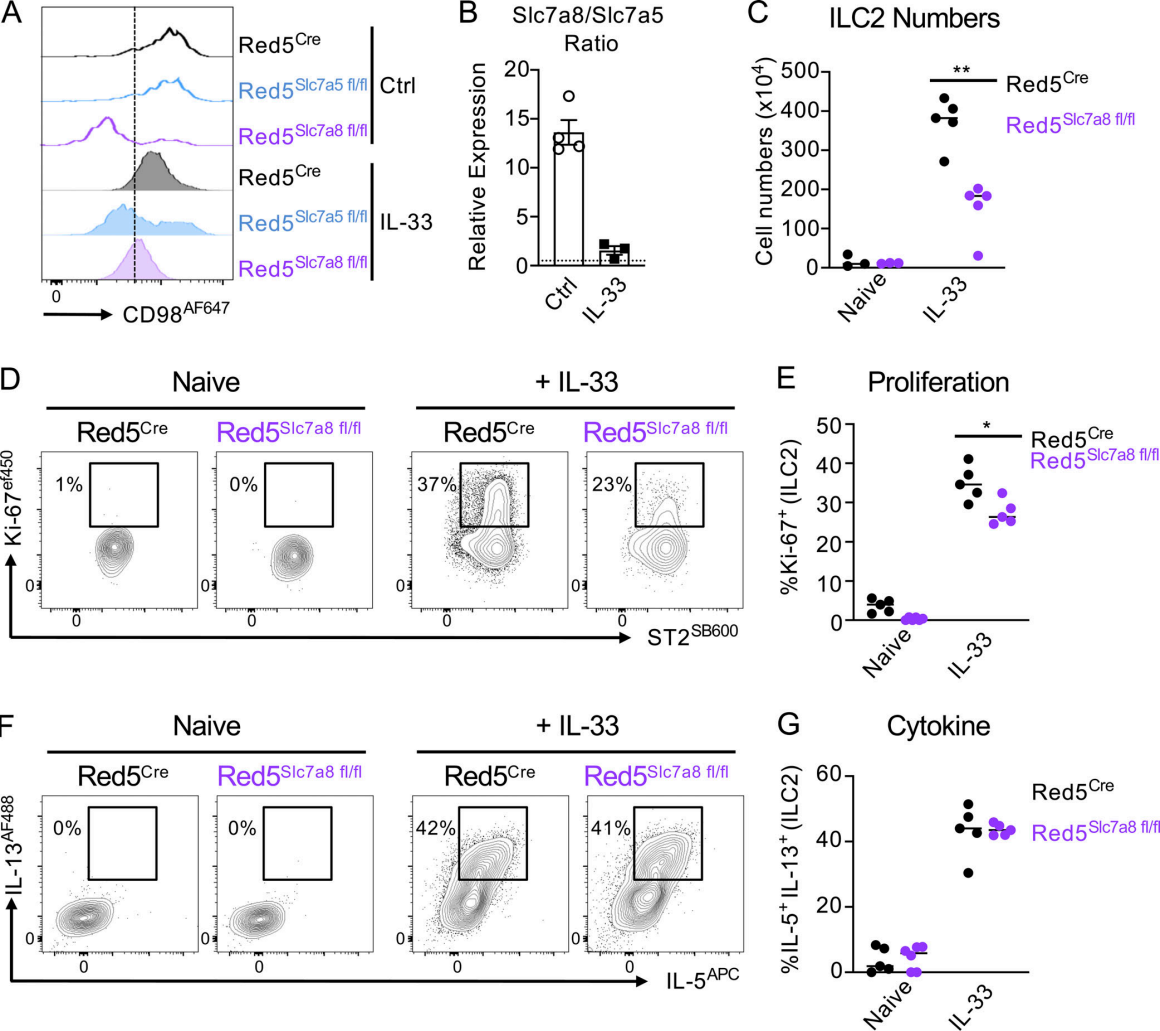
**Differential expression of *Slc7a8*/LAT2 is required for optimal ILC2 expansion following activation. (A)** Representative histograms of CD98 expression in lung ILC2 from control (Ctrl) and IL-33–treated Red5^Cre^ control, Red5^Cre^ x *Slc7a5*^fl/fl^, and Red5^Cre^ x *Slc7a8*^fl/fl^ mice (representative of *n* = 3–5 mice per group and at least two independent experiments). **(B)** Relative expression ratio of Slc7a8 to Slc7a5 in sort-purified ILC2 from control or IL-33–treated animals (*n =* 3–4 technical replicates per group, representative of two independent experiments). **(C–E)** (C) ILC2 numbers, (D) representative flow cytometry plots, and (E) quantification of Ki-67^+^ ILC2. **(F and G)** (F) Representative flow cytometry plots and (G) quantification of IL-5^+^ IL-13^+^ ILC2 in lung ILC2 from control (Ctrl) and IL-33–treated Red5^Cre^ control and Red5^Cre^ x *Slc7a8*^fl/fl^ mice. C–G, *n* = 3–5 mice per group and representative of at least two independent experiments. ILC2 gated as in Fig. S3 A. Data shown as individual values or mean ± SEM. Statistical tests performed: (C and E) one-way ANOVA with multiple comparisons. * P < 0.05, ** P < 0.01.

In contrast to *Slc7a5*, which has previously been attributed roles in the activation of other lymphocyte populations (Loftus et al., 2018; Sinclair et al., 2013), a role for *Slc7a8* in immune cell populations has not been widely reported and is poorly understood. Of note, however, *Slc7a8* was previously listed amongst the top signature-defining genes of intestinal ILC2 by RNA seq (Robinette et al., 2015) and was confirmed by RT-PCR to be highly and uniquely expressed in ILC2 derived from multiple tissues (Fig. S3 B), but not in resting T or B cells (Fig. S3, B and C), suggesting ILC2 may utilize multiple amino acid transporters to ensure a sufficient supply of these metabolic substrates. To test the role of *Slc7a8* in ILC2 responses, we obtained and validated a *Slc7a8* knockout allele (Fig. S3 D), which was subsequently converted to a loxP-flanked conditional allele via use of a FlpO recombinase. The Slc7a8^fl/fl^ allele was further backcrossed with Red5^Cre^ mice to generate animals with an ILC2-intrinsic deletion of *Slc7a8* (Red5^Slc7a8 fl/fl^). In contrast to our observations with IL-33–activated ILC2 (Fig. 3 B) and suggestive of a complimentary nature of these two amino acid transporters, we failed to observe any reduction in ILC2-associated surface CD98 at steady state in the absence of *Slc7a5* alone, whereas deletion of *Slc7a8* led to a marked reduction of surface CD98 in naive ILC2 (Fig. 4 A). However, confirming our previous findings upon IL-33 activation in vivo (Fig. 3 B), CD98 expression on ILC2 was markedly reduced by the absence of *Slc7a5*, whereas in the absence of *Slc7a8*, CD98 expression was in part maintained on the surface of ILC2 (Fig. 4 A). To try and reconcile these findings, we determined the relative expression of the two transporter genes in naive and IL-33–treated ILC2 and found that indeed *Slc7a8* was dominant in naive cells but that the ratio between the two CD98 partner chains became relatively equal after activation (Fig. 4 B), suggestive of different contributions of *Slc7a5* and *Slc7a8* to functional amino acid transporter heterodimers in activated and resting ILC2. Nonetheless, ILC2 frequencies and numbers were found to be comparable in naive Red5^Slc7a8 fl/fl^ and littermate control animals. Instead, a reduced expansion of lung ILC2 in response to IL-33 was observed in the absence of *Slc7a8* that was associated with a reduced frequency of Ki-67–expressing cells, but no cell-intrinsic defect in cytokine production was detected (Fig. 4, C–G), comparable with results obtained with *Slc7a5* deletion. Similar observations were also made in the mesenteric lymph node (Fig. S3, E and F). Thus, together, these findings suggest that ILC2 express distinct large amino acid transporters with differing expression patterns in resting and activated cells, both of which contribute to support optimal cell expansion upon activation.

### Amino acid transporter deficiency impairs ILC2 responses to helminth infection

**Figure 5.**
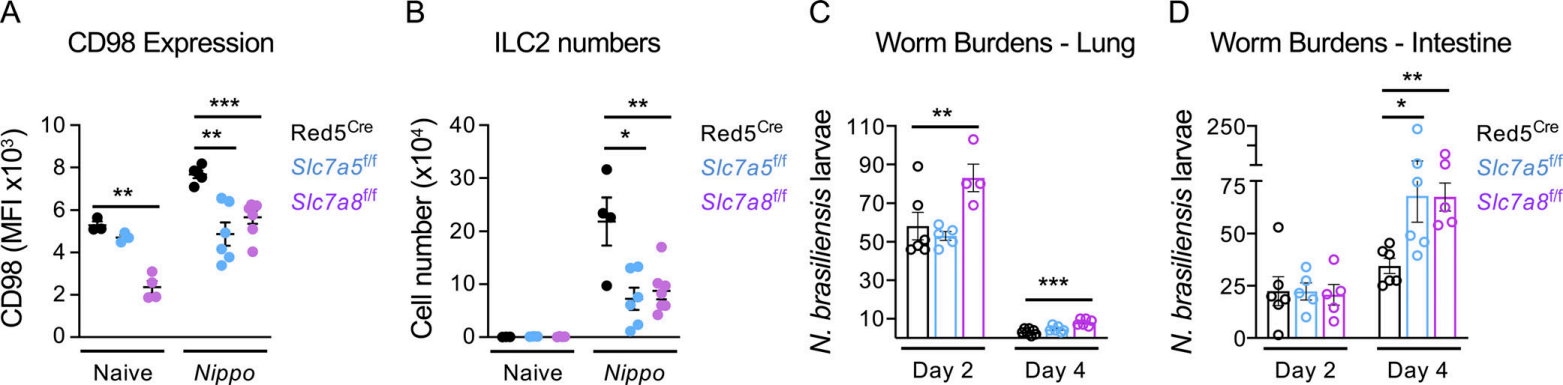
**Disruption of large neutral amino acid transport in ILC2 dampens protection to *N. brasiliensis* infection. (A and B)** (A) MFI of CD98 expression in lung ILC2, (B) ILC2 cell numbers from control naive and *N. brasiliensis*–infected (day 7 post infection [p.i.]) Red5^Cre^ x *Slc7a5*^fl/fl^ and Red5^Cre^ x *Slc7a8*^fl/fl^ mice (*n* = 3–6 mice per group and representative of two independent experiments). **(C and D)** Worm burdens in the (C) lung and (D) small intestine of *N. brasiliensis*–infected (day 2 and 4 p.i.) Red5^Cre^ controls, Red5^Cre^ x *Slc7a5*^fl/fl^, and Red5^Cre^ x *Slc7a8*^fl/fl^ mice (*n* = 4–6 mice per group and representative of two independent experiments). ILC2 gated as in Fig. S3 A. Data shown as individual values or mean ± SEM. Statistical tests performed: (A–D) one-way ANOVA with multiple comparisons. * P < 0.05, ** P < 0.01, *** P < 0.001.

**Figure S4.**
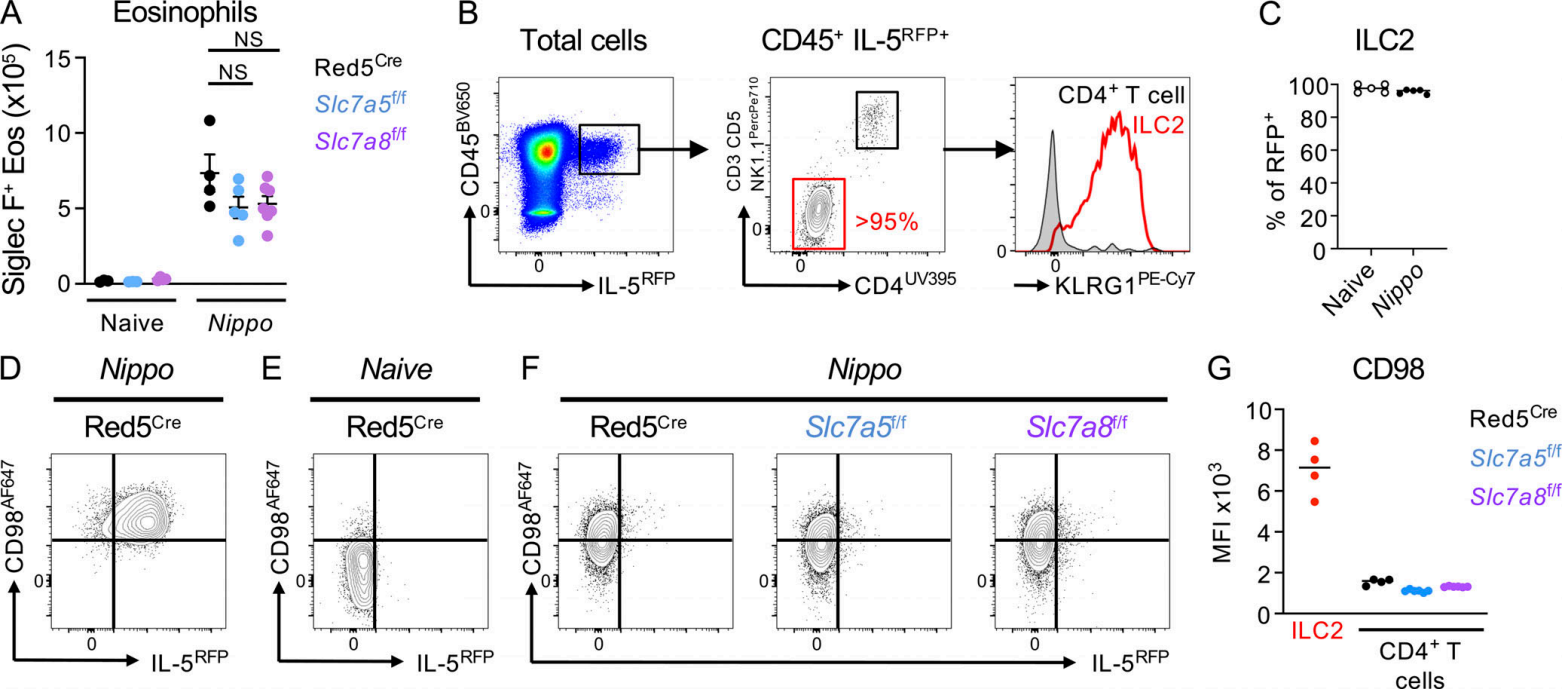
**Red5**^**Cre**^ **targeting of ILC2 and T cells during *N. brasiliensis* infection. (A)** Eosinophil quantification from control naive and *N. brasiliensis*–infected (day 7 post infection [p.i.]) Red5^Cre^ x *Slc7a5*^fl/fl^ and Red5^Cre^ x *Slc7a8*^fl/fl^ mice. **(B and C) (B)** Representative flow plots of IL-5^RFP+^ cell phenotype and (C) frequency of ILC2 within RFP^+^ cell population in the lung of *N. brasiliensis*–infected (day 7 p.i.) mice. **(A–C)** *n* = 3–6 mice per group and representative of two independent experiments. **(D–G)** Expression of CD98 and IL-5 RFP on (D) *N. brasiliensis* (*Nippo*)–infected lung ILC2, (E) naive lung CD4^+^ T cells from Red5^Cre^ mice, and (F) lung CD4^+^ T cells from Red5^Cre^, Red5^Cre^ x *Slc7a5*^fl/fl^, and Red5^Cre^ x *Slc7a8*^fl/fl^ mice of *Nippo* infected (day 7 post infection [p.i.]) mice. **(G)** Quantification of CD98 expression in ILC2 versus CD4^+^ T cells from Red5^Cre^, Red5^Cre^ x *Slc7a5*^fl/fl^, and Red5^Cre^ x *Slc7a8*^fl/fl^ mice of *Nippo*-infected (day 7 p.i.) mice. **(D–G)** All data *n* = 3–6 mice per group and representative of two independent experiments. Statistical tests performed: (A) one-way ANOVA with multiple comparisons.

Our data demonstrate a reduced ability of ILC2 to expand and proliferate in response to IL-33 in the absence of either *Slc7a5* or *Slc7a8*. To determine the role of these transporters in generating ILC2 response to a more physiological stimulus, we infected control Red5^Cre^, Red5^Slc7a5 fl/fl^, and Red5^Slc7a8 fl/fl^ mice with *N. brasiliensis* (Fig. 5). In line with our prior findings, we noted that while naive ILC2 had impaired surface CD98 expression in the absence of *Slc7a8*, they increased compensatory solute carrier expression upon activation by helminth infection (Fig. 5 A). In contrast, naive ILC2 lacking *Slc7a5* exhibited comparable CD98 surface expression but were unable to maintain surface CD98 upon activation by infection—again suggesting *Slc7a5* is proportionally increased and constitutes an elevated proportion of LAT heterodimers following ILC2 activation (Fig. 5 A). As with IL-33 activation, ILC2 expansion was reduced in the absence of either amino acid transporter following helminth infection (Fig. 5 B), and associated with trends toward reduced eosinophilia, although these failed to reach statistical significance (Fig. S4 A). Moreover, this reduced ILC2 response correlated with altered kinetics of infection. In particular, elevated worm burdens were observed in the intestines of mice at day 4 after infection as compared with control animals (Fig. 5, C and D). Notably, only mice lacking ILC2-intrinsic *Slc7a8* showed alterations in lung worm burdens at day 2, potentially suggesting a dominant role for this transporter in the early phase of an ILC2 response. As Red5^Cre^ is not specific to only ILC2 and can also target Th2 cell responses elicited by helminth infection, we assessed IL-5^RFP^ expression in CD4^+^ T cells at day 7 after infection but observed at this time point over 95% of RFP^+^ cells were ILC2 (Fig. S4, B and C), while surface CD98 expression was unaffected on T cells in floxed animals (Fig. S4, D–G). Thus, in the context of mucosal helminth infection, ILC2-intrinsic expression of the amino acid transporters LAT1 and/or LAT2 is required for optimal effector immune responses.

### Perturbation of ILC2 amino acid transport results in metabolic stress

**Figure S5.**
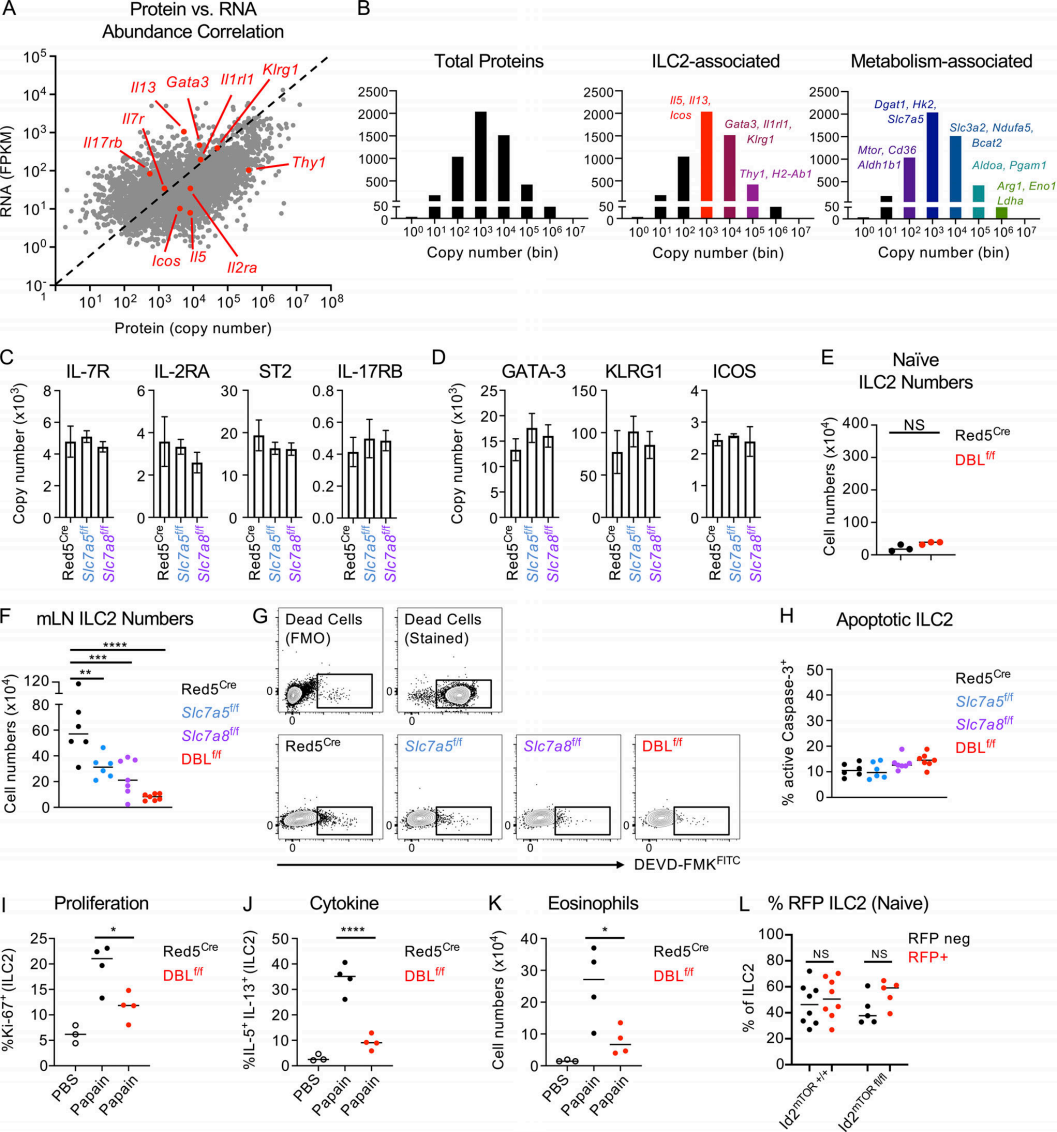
**Proteomic and phenotyping analysis of ILC2 lacking *Slc7a5, Slc7a8*, or both transporters. (A)** Comparison of relative mRNA expression and protein copy number in lung ILC2, analyzed by bulk RNA seq and proteomics, respectively. **(B)** Analysis of total protein copy number, and relative enrichment of key ILC2- and metabolism-associated proteins by copy number, in IL-33 elicited sort-purified lung ILC2 analyzed by proteomics. **(A and B)** *n =* 4 technical replicates pooled from five individual mice. **(C and D)** Proteomic analysis of sort-purified lung ILC2 from IL-33–treated Red5^Cre^ controls, Red5 x *Slc7a5*^fl/fl^, or Red5 x *Slc7a8*^fl/fl^ mice. (C) ILC and (D) ILC2 associated protein copy numbers (*n* = 3–4 replicates of cells pooled from two to three mice treated with IL-33, representative of a single experiment). **(E)** Cell numbers of lung ILC2 in naive Red5^Cre^ versus Red5 x *Slc7a5*^fl/fl^ x *Slc7a8*^fl/fl^ (DBL^f/f^). *n =* 3 and representative of two independent experiments. **(F)** Cell numbers of mLN ILC2 from IL-33–treated Red5^Cre^ controls, Red5 x *Slc7a5*^fl/fl^, Red5 x *Slc7a8*^fl/fl^, or Red5 x DBL^f/f^ mice. **(G and H)** (G) Representative flow cytometry plots and (H) quantification of active Caspase-3 (^FITC^DEVD-FMK) from lung IL-33–treated Red5^Cre^ controls, Red5 x *Slc7a5*^fl/fl^, Red5 x *Slc7a8*^fl/fl^, or Red5 x DBL^f/f^ mice. F–H representative of pooled data of *n =* 6–8 mice per group from at least two independent experiments. **(I–K)** Red5^Cre^ and Red5 x DBL^f/f^ mice were treated with three doses of 15 μg Papain, or PBS (DBL^f/f^), intranasally on day 1, 2, and 3 and euthanized at day 6. Expression of (I) Ki-67 and (J) IL-5 and IL-13 were assessed in lung ILC2, and (K) numbers of lung eosinophils were quantified (*n =* 3–4 per group, representative of two independent experiments). **(L)** quantification of RFP expression in naive lung ILC2 from Id2^ERT2Cre^ x Rosa26^tdRFP^ controls (Id2^mTOR+/+^) or Id2^ERT2Cre^ x Rosa26^tdRFP^ x mTOR^fl/fl^ mice (Id2^mTORfl/fl^; *n* = 5–8 per group, pooled from two independent experiments). Data shown as individual values or mean ± SEM. Statistical tests performed: (F and I–L) one-way ANOVA with multiple comparisons; (E) unpaired *t* test. * P < 0.05, ** P < 0.01, *** P < 0.001, **** P < 0.0001.

**Figure 6.**
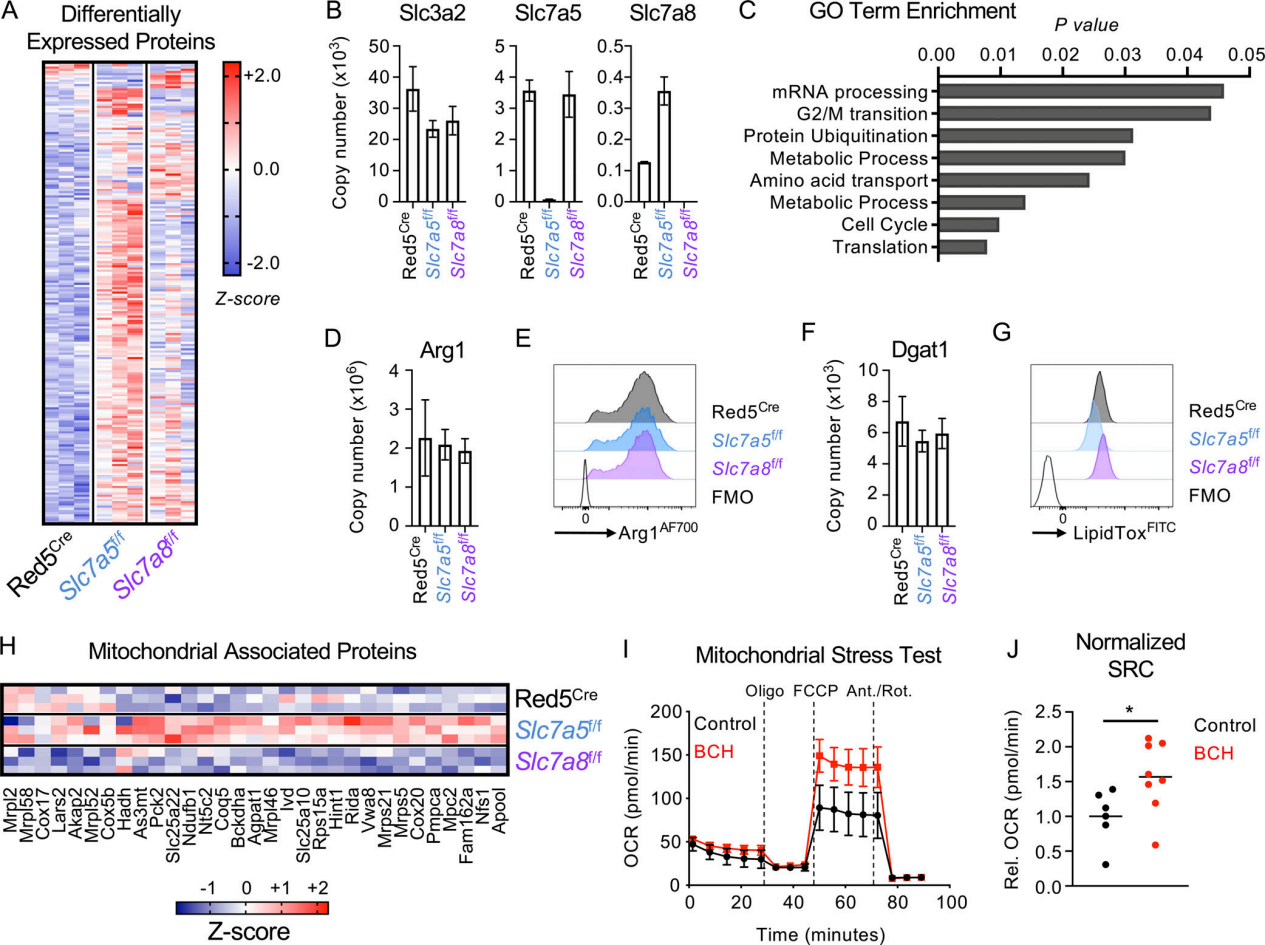
**Proteomics of LAT-deficient ILC2 reveals metabolic imbalance. (A and B)** (A) Differentially expressed proteins and (B) copy numbers of target amino acid transporter associated proteins in sort-purified lung ILC2 from IL-33–treated Red5^Cre^ controls, Red5 x *Slc7a5*^fl/fl^, or Red5 x *Slc7a8*^fl/fl^ mice (*n* = 3 replicates of cells pooled from two to three mice and representative of a single experiment). **(C)** Gene Ontology (GO) Term enrichment analysis of differentially expressed proteins across all genotypes in A. **(D and E)** (D) Arg1 protein copy number and (E) flow cytometry analysis in lung ILC2 of IL-33–treated mice. **(F and G)** (F) Dgat1 copy number and (G) intracellular lipid content (LipidTox staining) analyzed by flow cytometry analysis in lung ILC2 of IL-33–treated mice. D–F, *n* = 3 replicates of cells pooled from two to three mice and representative of a single experiment. E and G representative of at least *n = 3* per genotype and two independent experiments. **(H)** Enrichment of mitochondrial-associated proteins amongst differentially expressed proteins (identified with MitoCarta and MitoMiner). **(I and J)** (I) Extracellular flux analysis and (J) spare respiratory capacity (SRC) of sort-purified ILC2 from IL-33–treated mice cultured with or without 10 mM BCH overnight (*n* = 3–4 technical replicates per experiment, representative of two independent experiments; J data pooled from two independent experiments). ILC2 sorted and gated as in Fig. S3 A. Data shown as individual values or mean ± SEM. Statistical tests performed: (J) unpaired *t* test. * P < 0.05.

Ensuring a sufficient intracellular supply of amino acids is critical for cellular function not only by providing the building blocks for the generation of biomass but also via effects on cellular metabolism. Thus, we hypothesized that the consequences of perturbed amino acid transport would most likely be evident at the level of the proteome. To our knowledge, proteomic analysis has not previously been attempted on ILC populations; therefore, as a proof of concept, we first sort-purified wild-type ILC2 from IL-33 treated animals to determine feasibility. Using this approach, we were able to reproducibly detect over 5,000 individual proteins from ILC2. Comparison of protein copy number with bulk RNA seq data (Immgen) of mRNA transcripts revealed a largely linear correlation between genes and their products, including classical ILC2 genes and proteins (Fig. S5 A). However, in some cases (e.g., Thy1) the protein copy number diverged significantly from the relative gene expression level, indicating possible differences between transcriptomic and proteomic data in predicting ILC2 biology (Fig. S5 A). Proteins associated with ILC2 phenotype and function or cellular metabolism were robustly detected but varied in their total copy number distribution (Fig. S5 B). While these data demonstrate the feasibility of proteomic analysis of in vivo expanded ILC2, we were unable to generate sufficient material from naive animals for comparison. Next, to investigate the role of amino acid transporter deletion on the ILC2 proteome, we similarly sort-purified ILC2 from IL-33 treated Red5^Cre^, Red5^Slc7a5 fl/fl^, and Red5^Slc7a8 fl/fl^ mice and identified over 6,000 proteins, of which ∼200 proteins differed significantly by genotype (Fig. 6 A). We confirmed efficient deletion of Slc7a5 and Slc7a8 protein in the respective knockout animals (Fig. 6 B), while ILC2 expression of activating cytokine receptors (Fig. S5 C), transcription factors, and canonical surface markers (Fig. S5 D) were unchanged by transporter deletion. Performing Gene Ontology Term enrichment of the differentially expressed protein list, we identified cell cycle progression, metabolism, and protein translation as the major pathways perturbed in the absence of either amino acid transporter (Fig. 6 C).

Efficient nutrient uptake by ILC2 has previously been shown to act as a key determinant of cellular metabolism and the magnitude of the effector function, thus we investigated previously reported metabolic pathways implicated in the ILC2 response. However, we found that abundance of Arginase-1 (Arg1) protein was not altered in the absence of amino acid transporter expression (Fig. 6, D and E; Monticelli et al., 2016) nor was that of Diacylglycerol acyltransferase 1 (Dgat1; Fig. 6 F) or overall intracellular lipid storage (Fig. 6 G; Karagiannis et al., 2020). In contrast, we identified protein signatures indicative of altered mitochondrial biology—especially in the absence of Slc7a5—suggesting that lack of amino acid uptake may alter mitochondrial function of activated ILC2s (Fig. 6 H). To test this, we sort-purified wild-type ILC2 from IL-33 treated animals and cultured them overnight with the LAT1/2-inhibitor BCH to impede LAT-dependent uptake of amino acids and subsequently assessed the consequences via a mitochondrial stress test. We consistently observed that ILC2 incubated with BCH exhibited a higher oxygen consumption rate upon addition of carbonyl cyanide-p-trifluoromethoxyphenylhydrazone (FCCP), which disrupts mitochondrial proton transport and ATP synthesis (Fig. 6 I), and had an increased spare respiratory capacity compared with control cells (Fig. 6 J), together suggesting that amino acid transporter blockade may lead to altered mitochondrial function, possibly as a compensatory measure in the context of perturbed intracellular amino acid availability.

### Concurrent loss of *Slc7a5* and *Slc7a8* prevents optimal ILC2 effector responses

**Figure 7.**
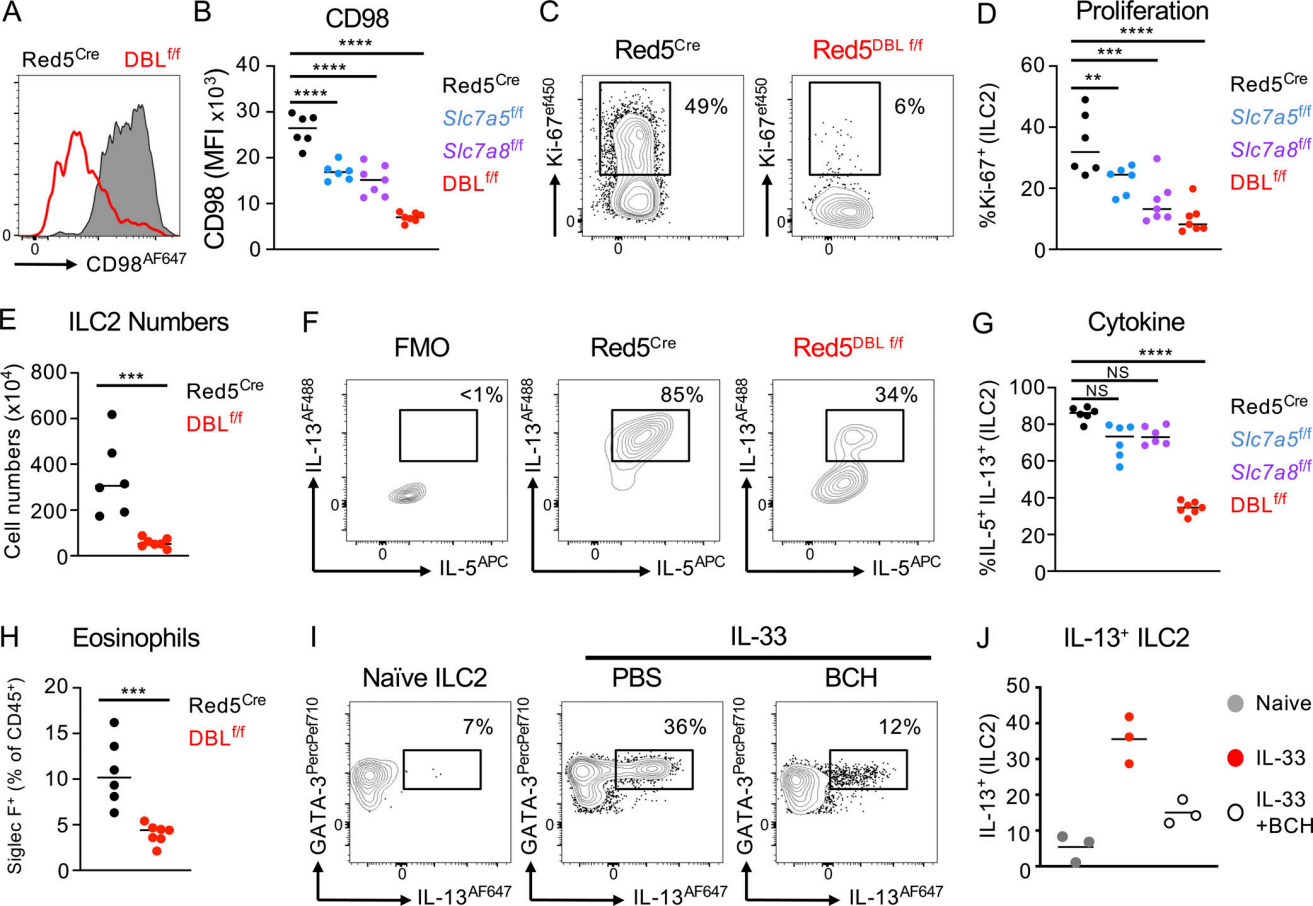
**Compound deletion of *Slc7a5* and *Slc7a8* reduces ILC2 effector responses. (A and B)** (A) Representative expression of CD98 and (B) quantification of CD98 MFI on lung ILC2 from IL-33–treated Red5^Cre^ controls, Red5 x *Slc7a5*^fl/fl^, Red5 x *Slc7a8*^fl/fl^, or Red5 x *Slc7a5*^fl/fl^ x *Slc7a8*^fl/fl^ (DBL^f/f^). **(C–E)** (C) Representative flow cytometry plots and (D) quantification of Ki-67 + ILC2, and (E) ILC2 numbers in control and DBL^f/f^ mice. **(F and G)** (F) Representative flow cytometry plots and (G) quantification of IL-5+ IL-13 + ILC2 in lung ILC2. **(H)** Lung Live CD45^+^ CD3^−^ CD5^−^ NK1.1^−^ B220^−^ Ly6C^−^ Ly6G^−^ CD11c^−^ Siglec F^+^ Eosinophil numbers in control and DBL^f/f^ mice. **(A–H)** All data representative of pooled data of *n =* 6–8 mice per group from at least two independent experiments. **(I and J)** (I) Representative flow cytometry plots, and (J) quantification of IL-13 expression of naive and IL-33 activated lung ILC2 in the presence or absence of BCH. Data are representative of two independent experiments with *n =* 3 replicates per culture condition. ILC2 gated as in Fig. S3 A. Data shown as individual values or mean ± SEM. Statistical tests performed: (B, D, and G) one-way ANOVA with multiple comparisons; (E and H) unpaired *t* test. ** P < 0.01, *** P < 0.001, **** P < 0.0001.

Our data suggested ILC2 express two distinct large neutral amino acid transporters, which are differentially expressed in resting and activated cells, but may have an overlapping function. While deletion of either transporter resulted in reduced ILC2 expansion, the remaining cells surprisingly retained the capacity to produce effector cytokine and exhibited only partial loss of proliferative capacity (Figs. 3 and 4). We hypothesized that redundancy between the two transporters could in part limit the effect on ILC2 responses by providing a degree of amino acid transport in the absence of either individual transporter chain. To address this hypothesis, we generated mice in which ILC2 lacked both *Slc7a5* and *Slc7a8* (Red5^Slc7a5/Slc7a8 fl/fl^; hereby referred to as DBL^f/f^; Fig. 7). Concurrent deletion of both transporters led to a compound decrease in surface CD98 expression on ILC2 beyond that observed with the deletion of either individual transporter (Fig. 7, A and B). Moreover, while DBL^f/f^ exhibited comparable ILC2 numbers in the lung at steady state when compared with control animals (Fig. S5 E), activation of ILC2 by IL-33 treatment revealed a near complete loss of proliferative capacity in ILC2 lacking both transporters and a marked defect in ILC2 expansion in the lungs (Fig. 7, C–E) and mesenteric lymph node (mLN; Fig. S5 F). Reductions in ILC2 numbers were not associated with increased apoptosis, as measured by active-Caspase 3 (Fig. S5, G and H), again suggestive of a defect in proliferation in the absence of one or both large neutral amino acid transporters. Strikingly, and in contrast to ILC2 deficiency in only a single amino acid transporter chain, DBL^f/f^ were observed to have a significantly reduced capacity to produce the effector cytokines IL-5 and IL-13 in response to IL-33 (Fig. 7, F and G), and the airways of these animals contained fewer eosinophils (Fig. 7 H). Similarly, IL-13 production of ILC2 from IL-33 treated wild-type was also reduced if both transporters were inhibited pharmacologically with BCH (Fig. 7, I and J). Similar findings were also observed when airway ILC2 responses were elicited with Papain (Fig. S5, I–K). Together, these findings suggest that only combined deficiency of *Slc7a5* and *Slc7a8* is sufficient to ablate both ILC2 proliferative and cytokine-producing capacity, highlighting the fundamental importance of amino acid uptake in facilitating ILC2 responses.

### Amino acid uptake regulates proliferation in part via mTOR

**Figure 8.**
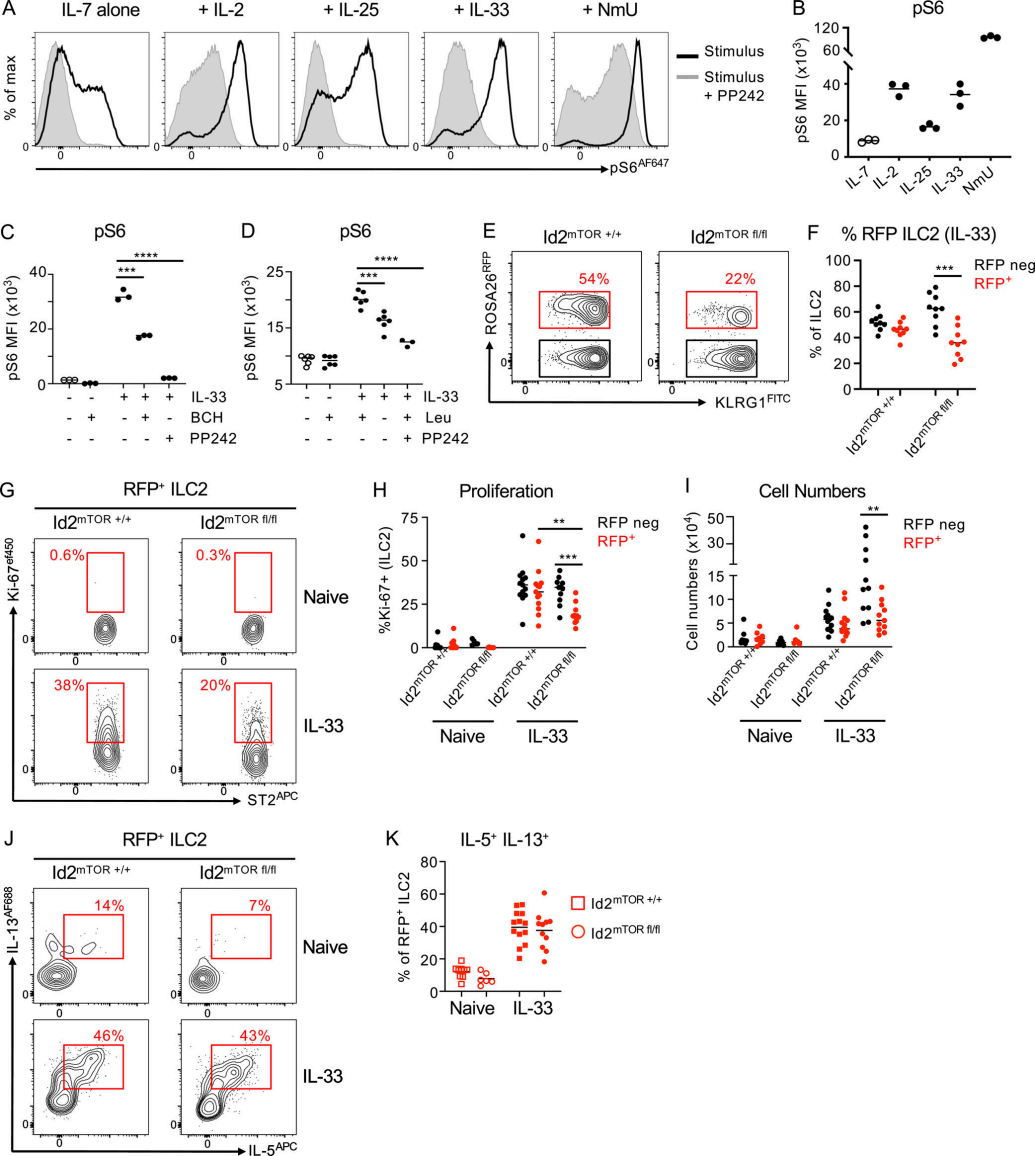
**Regulation of mTOR activation by amino acid transport controls magnitude of ILC2 response. (A and B)** (A) Representative flow plots and (B) quantification of pS6 in sort-purified ILC2 cultured for 30 min in the presence of 20 ng/ml IL-7 alone or IL-7 in combination with 20 ng/ml IL-2, IL-25, IL-33, or 1 μg/ml NmU, with or without the mTOR inhibitor PP242 (500 nm). *n* = 3 technical replicates per condition, representative of two independent experiments. **(C and D)** Phosphorylation of S6 in sort-purified ILC2 cultured with either (C) IL-33 with or without a 2 h pre-incubation with 10 mM BCH (*n* = 3 technical replicates per condition, representative of two independent experiments), or (D) IL-33 in ILC2 cultured with either Leucine sufficient or deficient media (*n* = 3–6 technical replicates per condition, representative of two independent experiments). **(E and F)** (E) Representative flow plots and (F) quantification of RFP expression in IL-33 elicited lung ILC2 from Id2^ERT2Cre^ x Rosa26^tdRFP^ controls (Id2^mTOR+/+^) or Id2^ERT2Cre^ x Rosa26^tdRFP^ x mTOR^fl/fl^ mice (Id2^mTORfl/fl^; *n* = 9 per group, pooled from two independent experiments). **(G–I)** (G) Representative flow plots, (H) quantification of Ki-67 expression, and (I) cell numbers of RFP^−^ and RFP^+^ lung ILC2 from naïve and IL-33–treated Id2^mTOR+/+^ and Id2^mTORfl/fl^ mice (*n* = 5–9 per group for naive mice and *n* = 11–13 for IL-33–treated mice, pooled from three independent experiments). **(J and K)** (J) Representative flow plots and (K) quantification of IL-5 and IL-13 expression in RFP^+^ ILC2 from naive and IL-33–treated Id2^mTOR+/+^ and Id2^mTORfl/fl^ mice (*n* = 5–9 per group for naive mice and *n* = 11–13 for IL-33–treated mice, pooled from three independent experiments). Data shown as individual values or mean ± SEM. Statistical tests performed: (C and D) one-way ANOVA with multiple comparisons; (F, H, and I) Kruskal–Wallis Test with multiple comparisons. ** P < 0.01, *** P < 0.001, **** P < 0.0001.

LAT-dependent intracellular amino acid availability has been extensively demonstrated to be a key regulator of activation of the mTOR. mTOR in turn acts as a critical cellular hub that integrates nutrient availability with activating signals from growth factors, cytokines, and other activating cues to determine downstream changes in cellular metabolism, protein translation, biomass synthesis, and proliferation (Jewell et al., 2013; Jones and Pearce, 2017; Kelly and Pearce, 2020). To first determine the cues that activate mTOR in ILC2 under normal culture conditions, we cultured sort-purified cells with cytokines, alarmins, and neuropeptides known to influence ILC2 responses. As expected, we found that IL-7 poorly induced mTOR activation, as indicated by phosphorylation of ribosomal protein S6 (pS6), consistent with its role in homeostatic maintenance of ILCs (Fig. 8 A). In contrast, ILC2 cultured with IL-2, IL-25, IL-33, and Neuromedin U (NmU) all drove robust phosphorylation of S6, which could be completely or partially prevented by co-incubation with the mTOR inhibitor PP242 (Fig. 8, A and B). We then determined whether ablation of amino acid uptake with the combined LAT1/LAT2-inhibitor BCH could alter mTOR activation in response to an activating signal (IL-33) and indeed found that pre-incubation of ILC2 with BCH reduced pS6 in the presence of IL-33 in comparison to cells activated with IL-33 alone, although pS6 was only partially suppressed when compared to complete mTOR inhibition with PP242 (Fig. 8 C). Similarly, ILC2 cultured in leucine-free media exhibited lower pS6 expression in response to IL-33 when compared with cells cultured in leucine-replete media (Fig. 8 D). Together, these findings suggest amino acid uptake via LATs on ILC2 acts in part to tune mTOR activation.

Finally, as singular or combined deletion of *Slc7a5* and *Slc7a8* led to reduced expansion of ILC2, we determined to what extent mTOR regulation could contribute to this phenotype. To circumnavigate potential developmental defects caused by a constitutive deletion of such a key sensing hub, we generated an inducible ERT2 Cre-driven model of mTOR deletion under the control of the *Id2* locus (to predominantly target ILCs), which upon tamoxifen administration drove an RFP reporter allele as previously described (Fiancette et al., 2021; Withers et al., 2016). Animals were then crossed with mice containing flanking loxP sites in the mTOR allele to facilitate deletion (Id2^mTOR fl/fl^). Upon tamoxifen administration, the relative ratio of Cre-activated (RFP^+^) and RFP^−^ ILC2 in naive control and Id2^mTOR fl/fl^ mice were largely comparable (Fig. S5 L). In contrast, following in vivo IL-33 administration, we noted a reduced relative frequency of RFP^+^ to RFP^−^ ILC2 in Id2^mTOR fl/fl^ mice, as well as a relative reduction of RFP^+^ ILC2 in Id2^mTOR fl/fl^ mice when compared with RFP^+^ cells from Id2^mTOR+/+^ control mice, indicating mTOR-deficient ILC2 may be at a competitive disadvantage to mTOR-sufficient wild-type cells (Fig. 8, E and F). In line with this, we also observed an intrinsic defect in Ki-67 expression and proliferation amongst Cre-activated RFP^+^ cells from Id2^mTOR fl/fl^ when compared with otherwise mTOR-competent RFP^−^ cells in the same mice, or in comparison with RFP^+^ cells from Id2^mTOR +/+^ control mice (Fig. 8, G–I). In contrast, proliferation and cell numbers were not perturbed by Cre activation and RFP expression in mice lacking the floxed allele (Fig. 8, G–I). Unexpectedly, RFP^+^ cells lacking mTOR showed no defect in cytokine production following IL-33 activation when compared with those in control mice (Fig. 8, J and K). Thus, our findings suggest amino acid uptake via LATs may regulate the magnitude of the ILC2 expansion via tuning of mTOR activation, but that amino acid transporter control of cytokine production may occur via other mechanisms, such as via the provision of biomass for protein translation.

## Discussion

Collectively, the findings presented here suggest that ILC2 are prepoised for the uptake of amino acids from the tissue environment via the amino acid transporters Slc7a5 and Slc7a8, in order to fuel optimal proliferation and cell expansion upon activation. In line with the findings of another recent study (Panda et al., 2022), this poised state appears to be in part due to the preferential expression of *Slc7a8* in naive ILC2 at steady state. In contrast, we noted a relative increase of *Slc7a5* expression and associated dependence on *Slc7a5* for surface CD98 following activation, similar to that reported for activated T cells (Sinclair et al., 2013). Notably, we found that near complete abrogation of both ILC2 proliferative capacity and effector cytokine production was only achieved in the combined absence of both *Slc7a5* and *Slc7a8*, either via concurrent cell-intrinsic deletion or through pharmacological targeting of both transporters. Thus, these data suggest ILC2 may employ two distinct large neutral amino acid transporters, both prior to and following activation, which are required to ensure sufficient intracellular amino acid availability to fuel a rapid innate response.

Nonetheless, whether these two transporters may confer specific biological advantages to ILC2 or simply provide the required redundancy to meet the amino acid demands of resting versus activated ILC2 remains unclear. One possibility is that the differing substrate specificity of the two transporters facilitates differential uptake of amino acids. Indeed, *Slc7a8* has been suggested to have higher specificity for alanine (Rossier et al., 1999), an amino acid found to be the most highly enriched in sort-purified ILC2 (Fig. 2 A), and which has recently also been shown to regulate mTOR activation in addition to classical substrates such as leucine (Meng et al., 2020). Of note, our data suggests *Slc7a8*/LAT2 may preferentially act in a steady state setting; however, we found no differences in ILC2 frequency or numbers across tissues in naive animals. One possibility is that *Slc7a8* may determine metabolic tone or innate fitness of naive ILC2 to prime them for rapid proliferation upon activation, although due to the limitations in performing extensive molecular and cellular analysis of naive ILC2, we have been unable to investigate the different contributions of LAT1 and LAT2 in resting ILC2 further within the scope of this study. Nonetheless, and in line with our findings, a recent report similarly demonstrated that human ILC2 isolated from peripheral blood are also uniquely poised for amino acid uptake, and that ILC2 cultured with inhibitors of downstream pathways associated with amino acid metabolism exhibited reduced cellular fitness and proliferation (Surace et al., 2021). This highlights a conserved requirement for amino acid uptake in ILC2 across species. Further studies and refined methodologies are needed to definitively dissect the different contributions of *Slc7a5* and *Slc7a8* to rare immune cell population biology and metabolism.

Finally, we observed that helminth infections increase the abundance of essential amino acids within the feces, in line with a previous report (Houlden et al., 2015), potentially linking changes in environmental cues with the metabolic and proliferative capacity of the responding innate immune cell. It is tempting to speculate that ILC2 may express multiple amino acid transporters not only to ensure sufficient import of metabolic substrates required to underpin their rapid innate expansion and functionality but also to enhance their sensitivity to environmental changes associated with pathogens that likely acted as a key evolutionary pressure to drive the emergence of this arm of the immune system. It is increasingly clear that a broad range of microbial and dietary metabolites regulate the activation of ILC2 (Surace et al., 2021; Shi et al., 2021; Karagiannis et al., 2020; Wilhelm et al., 2016; Monticelli et al., 2016), and together with classical activating signals, such as alarmins and neuropeptides, nutrient and metabolite availability likely act as a further regulatory layer to tune the magnitude of the immune response within the tissue microenvironment and facilitate rapid innate immune responses.

## Materials and methods

### Mice

6–8-wk-old female C57BL/6 were purchased from Envigo. Red5^Cre^ (B6(C)-*Il5*^tm1.1(iCre)Lky/^J, stock number 030926, originally generated by Richard Locksley, University of California, San Francisco), Id2ERT2^Cre^ (B6.129S(Cg)-*Id2*^tm1.1(*Cre*/ERT2)Blh^/ZhuJ, stock number 016222, originally generated by Yuan Zhuang, Duke University), and mTOR^fl/fl^ mice (B6.129S4-*Mtor*^tm1.2Koz^/J, stock number 011009, originally generated by Sara Kozma, University of Cincinnati) were originally imported from Jackson Laboratories. ROSA26^tdRFP^ were originally a kind gift from Hans Joerg Fehling (Ulm University, Ulm, Germany), and *Slc7a5*^fl/fl^ mice (B6.129P2-*Slc7a5*^tm1.1Daca^/J) were a kind gift from Doreen Cantrell (University of Dundee, Dundee, UK). *Slc7a8*^fl/fl^ mice were generated by crossing C57BL/6N-*Slc7a8*^tm2a(EUCOMM)*Hmgu*^/BayMmucd mice with mice containing a FlpO recombinase allele to remove the *lacZ* and Neomycin cassettes (KOMP/MMRRC repository, stock number 041243-UCD, originally generated by Arthur Beaudet, Baylor College of Medicine), generating flanking loxP sites spanning the critical exon. For the generation of mice lacking both *Slc7a5* and *Slc7a8* (Red5^Cre^ DBL^fl/fl^), mice were intercrossed until homozygous for both loxP flanked alleles. All transgenic lines utilized in this study were on a C57BL/6 background. In some experiments, mice were fed a diet containing 21% protein or 5% protein for 2 wk prior to infection or subsequent manipulation. The diets were purchased from Envigo laboratories (TD. 140918 and TD. 140711). For activation of inducible Cre alleles, mice were orally gavaged 5 mg Tamoxifen in corn oil every 2–3 d for a period of 2 wk and rested 1 wk prior to further experimental manipulation. For transgenic animal studies, age- and sex-matched littermate controls were used within experiments where possible; mice were maintained at the University of Manchester under specific pathogen–free conditions, with water and chow provided ad libitum, with constant temperature, and a 12-h light and dark cycle. All experiments were performed under license of the U.K. Home Office and under approved protocols. All animal studies were ethically reviewed and carried out in accordance with Animals (Scientific Procedures) Act 1986 and the GSK Policy on the Care, Welfare, and Treatment of Animals.

### In vivo ILC2 activation

Mice were injected intraperitoneally with 0.5 μg of recombinant IL-33 (BioTechne) on days 0, 2, and 4 unless otherwise indicated. To maximize cell yield for sort-purification of ILC2 and ex vivo assays, mice received additional doses of IL-33 and/or a higher dosing regimen (1 μg). Alternatively, mice received 15 μg Papain in 40 μl PBS, or PBS alone, intranasally on days 1, 2, and 3 under transient isoflurane anesthesia and were euthanized on day 6.

### Helminth infections

Mice were infected with 300 L3 *N. brasiliensis* via subcutaneous injection or with 250 infective larvae of either *H. polygyrus* or *T. spiralis* via oral gavage. Helminth life cycles were maintained, and infective larvae were kindly provided by the groups of Judith Allen, Richard Grencis, and John Grainger at the University of Manchester.

### Tissue processing

Briefly, lungs were collected in 2 ml of PBS and thoroughly minced prior to the addition of 2 mg/ml collagenase D and 33 μg/ml DNase. Tissue was shaken at 37°C for 40 min at 200 rpm prior to mechanical disruption and passing over a 70-μm nylon filter and flushing of remaining tissue with PBS. Supernatants were pelleted and cells briefly incubated with 2 ml ACK buffer to lyse residual red blood cells, prior to being washed and resuspended in PBS containing 5% FCS and 1 mM EDTA for flow cytometry staining. mLNs were processed, in a similar manner without enzymatic digestion, via manual disruption over a 70-μm nylon filter. Intestinal lamina propria preparations were isolated by removing all fat and Peyer’s patch from intestines, opening longitudinally and flushing in PBS, followed by extensive vigorous vortexing of intestinal tissue in PBS, and subsequent rounds of incubation and constant shaking with PBS containing 5% FCS, 1 mM EDTA, and 1 mM dithiothreitol at 37°C to remove mucus and epithelium. The remaining tissue was then incubated with constant shaking at 37°C in RPMI media containing 0.1 mg/ml collagenase/dispase (Roche) and 20 μg/ml DNase (Sigma-Aldrich) for 45 min. Supernatant containing liberated lymphocytes was collected by passing tissue over a 70-μm nylon filter, and cells pelleted and resuspended in PBS containing 5% FCS and 1 mM EDTA for flow cytometry staining.

### Flow cytometry and cell sorting

Surface antibody staining was performed in PBS containing 5% FCS and 1 mM EDTA and using a Fixable Aqua Dead Cell (Invitrogen) to determine viability. Cells were stained with the following cell surface antibodies and using the conjugates indicated in the figure labels and utilized for analysis with a BD Fortessa or cell-sorting with a BD Aria Influx; CD127 (IL-7Rα, Brilliant Violet 421, PE, or FITC, clone A7R34; eBioscience), ST2 (IL-33R Biotin; clone RMST2-33; eBioscience), CD45 (brilliant violet 650; clone 30-F11; BioLegend), CD3 (PerCP-Cyanine 5.5 or PE/Cy7; clone 145-2C11), CD5 (PerCP-Cyanine 5.5 or PE/Cy7, clone 53-7.3; BioLegend) NK1.1 (PerPC- Cyanine 5.5, BV395, or PE/Cy7, clone PK136; eBioscience), CD90-2 (Alexa Fluor 700 AM; clone 30-H12; BioLegend), B220 (CD45R, APC-e Fluor 780, clone RA3-6B2; eBioscience), CD11b (super bright 600, APC-e Fluor 780; clone M1/70; Invitrogen), CD11c (APC-e Fluor 780, clone N418; eBioscience), CD4 (super bright 600, clone RM4-5; eBioscience; BV395, clone GK1.5; eBioscience), SA-APC (streptavidin APC, eBioscience), SA-SB600 (streptavidin super bright 600; eBioscience), CD98 (Alexa Fluor 647; clone RL388; BioLegend), CD8α (FITC; clone 53-6.7; BioLegend), KLRG1 (PeCyanine 7, Invitrogen, FITC, or Pe-eFlour 610, clone 2F1; eBioscience), and MHCII (eFluor 450; clone M5/114,15.2; Invitrogen). To facilitate detection of natural killer T cells, samples were stained with a PE-labeled tetramer (CD1d PBS-57 or unloaded control) provided by the National Institutes of Health Tetramer Core Facility.

Intracellular staining was performed by fixing cells for 30 min in FoxP3 fix/perm buffers (eBioscience) prior to staining for 30 min in permeabilization buffer (eBioscience) at 4°C. Alternatively, in order to retain reporter signals, cells were first fixed with BD Cytofix/Cytoperm buffer (BD Biosciences) for 1 h at 4°C prior to staining intracellular antigens overnight at room temperature. Intracellular antibodies utilized in this study RORγt (PE; clone B2D), Ki-67 (eFluor 450, clone SolA1s; eBioscience), GATA 3 (PercP eFluor 710, clone TWAJ; eBioscience), Arg1 (Alexa Fluor 700, clone A1exF5; eBioscience), IL-5 (PE or APC, clone TRFK5, BioLegend; eBioscience), and IL-13 (Alexa Fluor 488, or PeCyanine 7; Invitrogen; or eFluor 660, clone eBio13A; eBioscience). For phosphoFlow, stimulated cells were fixed with pre-warmed Phosflow Lyse/Fix buffer (BD Biosciences) for 10 min, washed and permeabilized with ice-cold Perm Buffer III (BD biosciences) for 30 min, and subsequently stained with pS6 (Ser235 Ser236; APC, clone cupk43k; eBioscience). The Kynurenine uptake assay was performed as previously described (Sinclair et al., 2018).

### RT-PCR and bulk RNA seq

Total RNA was purified using the RNeasy Micro Kit (Qiagen) and cDNA was prepared using the high-capacity cDNA reverse transcription kit (Applied Biosystems). Real-time quantitative PCR was performed for *Slc7a5* and *Slc7a8* with the real-time PCR StepOnePlus system (Applied Biosystems) and normalized to *Actb* (β Actin) housekeeping gene. Primers; *Actb* Fwd 5′-TCC​TAT​GTG​GGT​GAC​GAG-3′, *Actb* Rev 5′-CTC​ATT​GTA​GAA​GGT​GTG​GTG-3′, *Slc7a5* Fwd 5′-CTG​GAT​CGA​GCT​GCT-3′, *Slc7a5* Rev 5′-GTT​CAC​AGC​TGT​GAG​GAG​C-3′, *Slc7a8* Fwd 5′-AAG​AAG​CCT​GAC​ATT​CCC​CG-3′, *Slc7a8* Rev 5′-TGT​GTT​GCC​AGT​AGA​CAC​CC-3′. For bulk RNA seq of wild-type ILC2, RNA was isolated from sort-purified cells, as above, and library preparation and bulk RNA seq were performed commercially with Novogene (UK) Company Ltd. Briefly, and normalized RNA was used to generate libraries using NEB Next Ultra RNA library Prep Kit (Illumina). Indices were included to multiplex samples and mRNA was purified from total RNA using poly-T oligo-attached magnetic beads. After fragmentation, the first-strand cDNA was synthesized using random hexamer primers followed by second-strand cDNA synthesis. Following end repair, A-tailing, adaptor ligation, and size section libraries were further amplified and purified, and insert size validated on an Agilent 2100 and quantified using quantitative PCR. Libraries were then sequenced on an Illumina NovaSeq 6000 S4 flowcell with PE150 according to results from library quality control and expected data volume.

### RNA seq

Analysis of innate lymphoid cell transcriptional signatures was performed using previously published RNA seq data sets (Robinette et al., 2015). Data sets are available at http://www.immgen.org and via GEO accession number GSE109125.

### Extracellular flux analysis

ILC2 were sort-purified from IL-33–treated mice and incubated overnight with or without 10 mM BCH. Seahorse plates and cartridges were prepared 18 h before by adding 200 μl XF Calibrant to each well (Seahorse Bioscience/Agilent) to submerge probes and incubating at 37°C to calibrate. ILC2 were washed and plated onto poly-D-lysine–coated XF96 plates with XF RPMI media and rested for 30 min at 37°C prior to analysis. For the mitochondrial stress test, Seahorse medium was supplemented with 25 mM glucose (Thermo Fisher Scientific), 1 mM sodium pyruvate, and 2 mM L-glutamine (Sigma-Aldrich), and pH adjusted to 7.4. Cellular bioenergetics were assessed at 5-min intervals following sequential addition of 2 μM Oligomycin, 2 μM FCCP, 0.5 μM Antimycin A, and 0.5 μM Rotenone (all Sigma-Aldrich) using an XF96e extracellular flux analyzer (Seahorse Bioscience/Agilent) via sequential addition of 2 μM Oligomycin, 1.5 μM FCCP, 0.5 μM Antimycin A, and 0.5 μM Rotenone (all Sigma-Aldrich).

### Fecal metabolomics

The metabolic profiles of fecal samples were measured using ^1^H nuclear magnetic resonance (NMR) spectroscopy as previously described (Alcon-Giner et al., 2020). Briefly, fecal samples (30 mg) were defrosted and combined with 600 µl of water and zirconium beads (0.45 g). Samples were homogenized with a Precellys 24 instrument (45 s per cycle, speed 6,500, two cycles) and spun at 14,000 *g* for 10 min. The supernatants (400 µl) were combined with 250 µl phosphate buffer (pH 7.4, 100% D_2_O containing 3 mM NaN_3_, and 1 mM of 3-(trimethyl-silyl)-[2,2,3,3-^2^H4]-propionic acid [TSP] for the chemical shift reference at δ0.0) before vortexing and centrifugation at 14,000 *g* for 10 min and transfer to 5-mm NMR tubes. All samples were analyzed on a Bruker 700 MHz spectrometer equipped with a cryoprobe (Bruker Biospin) operating at 300 K. ^1^H NMR spectra were acquired for each sample using a standard one-dimensional pulse sequence using the first increment of the Nuclear Overhauser Enhancement pulse sequence for water suppression as previously described (Beckonert et al., 2007). Raw spectra were automatically phased, baseline-corrected, and calibrated to TSP using Topspin 3.2 (Bruker Biospin) and then digitized in a Matlab environment (Version 2018; Mathworks Inc) using in-house scripts. Redundant spectral regions (related to water and TSP resonance) were removed, and the spectral data were manually aligned and normalized to the probabilistic quotient using in-house Matlab scripts. Peak integrals (relating to relative abundance) for metabolites of interest were calculated for each sample.

### Proteomics and mass spectrometry (MS)

For initial establishment of proteomic methodology, technical replicate pools of 5 million ILC2 were sort-purified from IL-33–treated mice. For comparison of control and conditional knockout mice, technical replicates averaging between 500,000 and 1 million ILC2 pooled from two individual mice were sort-purified. Cell pellets were washed extensively with PBS to remove residual FCS and snap frozen. Samples were prepared for MS by adding 100 µl of lysis buffer (5% sodium dodecyl sulfate, 50 mM Triethylammonium bicarbonate, pH 8.5, and 10 mM Tris(2-carboxyethyl)phosphine) to each cell pellet and shaking at 1,000 rpm at room temperature for 5 min. Lysates were boiled for 5 min at 95°C, sonicated for 15 cycles of 30 s each, and treated with 1 µl benzonase for 15 min at 37°C. Protein yield was determined using the EZQ protein quantitation kit (Thermo Fisher Scientific) according to manufacturer’s instructions. Lysates were alkylated with 20 mM iodoacetamide for 1 h at room temperature in the dark. Protein lysates were loaded onto S-Trap micro columns (ProtiFi) following the manufacturer’s instructions. Proteins were digested with 20:1 protein:trypsin (Trypsin Gold, Promega) in 50 mM ammonium bicarbonate for 3 h at 47°C before adding an additional 1 µg of trypsin and digesting for a further 1 h at 47°C. Peptides were eluted from columns and dried by SpeedVac and resuspended in 1% formic acid at a peptide concentration of 0.1 µg/µl.

For liquid chromatography (LC)-MS analysis of wild-type ILC2, 1.5 µg of peptide for each sample was analyzed on a Q-Exactive-HF-X (Thermo Fisher Scientific) mass spectrometer coupled with a Dionex Ultimate 3000 RS (Thermo Fisher Scientific). The following LC buffers were used: buffer A (0.1% formic acid in Milli-Q water [vol/vol]) and buffer B (80% acetonitrile and 0.1% formic acid in Milli-Q water [vol/vol]). 1.5 μg aliquot of each sample was loaded at 15 μl/min onto a trap column (100 μm × 2 cm, PepMap nanoViper C18 column, 5 μm, 100 Å, Thermo Fisher Scientific) equilibrated in 0.1% TFA. The trap column was washed for 3 min at the same flow rate with 0.1% TFA and then switched in-line with a Thermo Fisher Scientific resolving C18 column (75 μm × 50 cm, PepMap RSLC C18 column, 2 μm, 100 Å). Peptides were eluted from the column at a constant flow rate of 300 nl/min with a linear gradient from 3% buffer B to 6% buffer B in 5 min, then from 6% buffer B to 35% buffer B in 115 min, and finally to 80% buffer B within 7 min. The column was then washed with 80% buffer B for 4 min and re-equilibrated in 3% buffer B for 15 min. Two blanks were run between each sample to reduce carryover. The column was kept at a constant temperature of 50°C at all times. Data were acquired using an easy spray source operated in positive mode with spray voltage at 1.9 kV, capillary temperature at 250°C, and funnel radiofrequency at 60°C. The MS was operated in data independent acquisition (DIA) mode using parameters previously described (Muntel et al., 2019), with some modifications. A scan cycle comprised a full MS scan (m/z range from 350 to 1,650, with a maximum ion injection time of 20 ms, a resolution of 120,000, and an automatic gain control [AGC] value of 5 × 106). MS survey scan was followed by MS/MS DIA scan events using the following parameters: default charge state of 3, resolution 30,000, maximum ion injection time 55 ms, AGC 3 × 106, stepped normalized collision energy 25.5, 27, and 30, and fixed first mass 200 m/z. Data for both MS and MS/MS scans were acquired in profile mode.

For conditional knockout LC-MS analysis, peptides were analyzed on a Q Exactive Plus Mass Spectrometer (Thermo Fisher Scientific) coupled to a Dionex Ultimate 3000 RS (Thermo Fisher Scientific). The following LC buffers were used: buffer A (0.1% formic acid in Milli-Q water [vol/vol]) and buffer B (80% acetonitrile and 0.1% formic acid in Milli-Q water [vol/vol]). An equivalent of 1.5 µg of each sample was loaded at 10 μl/min onto a µPAC trapping C18 column (Pharmafluidics). The trapping column was washed for 6 min at the same flow rate with 0.1% TFA and then switched in-line with a Pharma Fluidics, 200 cm, µPAC nanoLC C18 column. The column was equilibrated at a flow rate of 300 nl/min for 30 min. The peptides were eluted from the column at a constant flow rate of 300 nl/min with a linear gradient from 1% buffer B to 3.8% buffer B in 6 min, from 3.8% B to 12.5% buffer B in 40 min, from 12.5% buffer B to 41.3% buffer B within 176 min, and then from 41.3% buffer B to 61.3% buffer B in 14 min. The gradient was finally increased from 61.3% buffer B to 100% buffer B in 1 min, and the column was then washed at 100% buffer B for 10 min. Two blanks were run between each sample to reduce carryover. The column was kept at a constant temperature of 50°C.

Q Exactive Plus was operated in positive ionization mode using an easy spray source. The source voltage was set to 2.2 Kv and the capillary temperature was 275°C. Data were acquired in Data Independent Acquisition Mode as previously described (Doellinger et al., 2020) with some modifications. A scan cycle comprised of a full MS scan (m/z range from 345 to 1,155), resolution was set to 70,000, AGC target 3 × 10^6^, and maximum injection time 200 ms. MS survey scans were followed by DIA scans of dynamic window widths with an overlap of 0.5 Th. DIA spectra were recorded at a resolution of 17,500 at 200 m/z using an AGC target of 3 × 10^6^, a maximum injection time of 55 ms, and a first fixed mass of 200 m/z. Normalized collision energy was set to 25% with a default charge state set at 3. Data for both MS scan and MS/MS DIA scan events were acquired in profile mode.

Raw MS data were processed using Spectronaut (Biognosys; version 14.5.200813.47784 for wild-type ILC2 and version 14.10.201222.47784 for conditional knockout comparisons). For all searches, the DirectDIA option was selected. The following parameters were chosen: cleavage rules were set to Trypsin/P, maximum peptide length 52 amino acids, minimum peptide length 7 amino acids, maximum missed cleavages two, and calibration mode automatic. Carbamidomethylation of cysteine was set as a fixed modification while the following variable modifications were selected: oxidation of methionine, deamidation of asparagine and glutamine, and acetylation of the protein N-terminus. The false discovery rate threshold for both precursor and protein was set at 1%. DirectDIA data were searched against a mouse database from Uniprot release 2020 06. This database consisted of all manually annotated mouse SwissProt entries along with mouse TrEMBL entries with protein-level evidence and a manually annotated homolog within the human SwissProt database. Estimates of protein copy number per cell were calculated using the histone ruler method (Wisniewski et al., 2014).

Proteomic data sets are available from the ProteomeXchange data repository (PRIDE), accession numbers PXD038792 (IL-33 treated wild-type ILC2) and PXD038786 (IL-33 treated Red5^Cre^, Red5^Cre^ x Slc7a5^fl/fl^, and Red5^Cre^ x Slc7a8^fl/fl^ ILC2).

### Statistics

Data are presented as mean ± SEM, unless indicated otherwise. Statistical analyses were performed using either unpaired *t* test, Mann–Whitney test, Kruskal–Wallis test, or one-way ANOVA with multiple comparisons, as indicated in the figure legend.

### Online supplemental material

Fig. S1 demonstrates changes in metabolite and dietary factors following helminth infection. Fig. S2 demonstrates amino acid transport in ILC2 and other tissue-resident lymphocytes. Fig. S3 details *Slc7a8* expression in ILC2 and validates deletion and phenotype in ILC2-conditional knockout mice. Fig. S4 provides supportive and contextual data regarding Red5 Cre specificity and Cre-driven phenotypes in the context of *N. brasiliensis* infection. Fig. S5 contains additional data from proteomic analyses and further phenotyping in mice lacking ILC2-intrinsic one or both amino acid transporters.
